# mRNA and circRNA mislocalization to synapses are key features of Alzheimer’s disease

**DOI:** 10.1371/journal.pgen.1011359

**Published:** 2024-07-29

**Authors:** Samuel N. Smukowski, Cassidy Danyko, Jenna Somberg, Eli J. Kaufman, Meredith M. Course, Nadia Postupna, Melissa Barker-Haliski, C. Dirk Keene, Paul N. Valdmanis

**Affiliations:** 1 Division of Medical Genetics, Department of Medicine, University of Washington, Seattle, WA, United States of America; 2 Department of Genome Sciences, University of Washington, Seattle, Washington, United States of America; 3 Fred Hutch Cancer Center, Basic Sciences Division, University of Washington, Seattle, Washington, United States of America; 4 Department of Laboratory Medicine and Pathology, University of Washington School of Medicine, Seattle, Washington, United States of America; 5 Department of Molecular Biology, Colorado College, Colorado Springs, Colorado, United States of America; 6 Department of Pharmacy, University of Washington School of Pharmacy, Seattle, Washington, United States of America; 7 Department of Neurological Surgery, University of Washington School of Medicine, Seattle, Washington, United States of America; University College London, UNITED KINGDOM OF GREAT BRITAIN AND NORTHERN IRELAND

## Abstract

Proper transport of RNAs to synapses is essential for localized translation of proteins in response to synaptic signals and synaptic plasticity. Alzheimer’s disease (AD) is a neurodegenerative disease characterized by accumulation of amyloid aggregates and hyperphosphorylated tau neurofibrillary tangles followed by widespread synapse loss. To understand whether RNA synaptic localization is impacted in AD, we performed RNA sequencing on synaptosomes and brain homogenates from AD patients and cognitively healthy controls. This resulted in the discovery of hundreds of mislocalized mRNAs in AD among frontal and temporal brain regions. Similar observations were found in an APP^swe^/PSEN1^dE9^ mouse model. Furthermore, major differences were observed among circular RNAs (circRNAs) localized to synapses in AD including two overlapping isoforms of *circGSK3β*, one upregulated, and one downregulated. Expression of these distinct isoforms affected tau phosphorylation in neuronal cells substantiating the importance of circRNAs in the brain and pointing to a new class of therapeutic targets.

## Author summary

Synaptic plasticity is the foundation for cognition and describes the process by which synaptic connections between neurons are formed, strengthened, or weakened based on signals transmitted by neighboring neurons. Essential to this process is the ability of synapses to respond rapidly to signals and restructure their morphology by translating proteins from a pool of localized mRNAs which is orchestrated by a complex cytoskeletal trafficking system. Given that synapse loss is a cardinal feature of Alzheimer’s disease (AD), we RNA sequenced fractionated synaptic particles from brain samples from AD patients and healthy controls to determine whether RNA localization to synapses is impacted in AD. We discovered hundreds of mislocalized mRNAs as well as circular RNAs (circRNAs) which are non-coding isoforms shown to play critical regulatory roles in the brain. Among our data, we discovered two isoforms of *circGSK3β*, one upregulated, and one downregulated in AD. Expression of these isoforms in neuronal culture was shown to affect tau phosphorylation, dysregulation of which is a hallmark of AD. These findings point to RNA localization and circRNAs as key components to understanding AD pathology and implicate new targets for AD therapies.

## Introduction

Alzheimer’s disease (AD) is the most prevalent form of dementia and estimated to account for at least 50 million cases worldwide [1]. The disease is characterized by extracellular accumulation of amyloid-β (Aβ) peptides into plaques and intracellular neurofibrillary tangles of hyperphosphorylated microtubule-associated protein tau (MAPT) [2]. AD can be separated into two types: familial (fAD) early-onset and late-onset sporadic (sAD) forms. Only 5% of cases are fAD, which are connected to pathogenic variants in amyloid precursor protein (*APP*), the originating protein for Aβ peptides, and presenilin 1 (*PSEN1*) and presenilin 2 (*PSEN2*). *PSEN1* and *PSEN2* are components of the gamma-secretase complex that processes APP and produces Aβ peptides [3]. Most cases are sAD and have modest genetic risk factors, apart from a risk allele in apolipoprotein E (*APOE*), called ApoE4 [4,5].

For years, AD research has centered upon the formation of amyloid plaques which is the most distinguishable event before downstream pathologies and cognitive impairment are observed [2]. Yet, it remains to be explained whether amyloid plaques themselves result in neurodegeneration or simply precipitate other events that ultimately cause cognitive dysfunction. This has led to several lines of research dedicated to specific hypotheses of AD mechanisms including neuroinflammation, tau neurofibrillary tangles, excitotoxicity, and metabolic dysfunction [6]. It has also been found that some patients are cognitively resilient to some of the characteristic pathologies making amyloid plaques necessary, but not sufficient, for AD dementia [2,7].

Synapse loss is one of the cardinal features of AD pathology and precedes Aβ deposition and tau neurofilament tangles. Therefore, maintaining synaptic integrity is important to prevent cognitive decline [8]. A key component of this integrity is the localized translation of mRNAs at the synapse in rapid response to stimuli that modify synaptic structure and connections that are essential for synaptic plasticity [9–11]. For localized translation to take place, mRNAs need to be properly trafficked to synaptic destinations. This is orchestrated by a combination of localization signals in the untranslated regions of mRNA and formation of complexes with RNA binding proteins (RBPs) and motor proteins that shuttle RNA-protein biomolecular condensates along microtubules in the neurites to reach synaptic destinations [12–23]. Given the importance and complexity of this process for cognition, it remains to be seen how RNA transport and synaptic-localized translation is impacted by AD pathologies. Previous research has shown that retrograde transport of transcriptional factors in neurons further propagates pathology throughout the brain [24]. Additional studies have revealed that cytoskeletal transport is impacted in AD including roles for SORL1 and Dyneins [25,26]. The observed dysfunctions of these and other proteins involved in cytoskeletal transport and the endocytic cycle has led to hypotheses describing that at least some of the downstream AD pathology is propagated by impaired vesicle transportation along the cytoskeleton causing a “traffic jam” along transportation conduits [27,28]. Given that RNA, proteins, and vesicles are transported along the same cytoskeletal network, competition may arise for transportation among all these cargos, and disruption of this critical system can impact cellular function as a whole. Thus, errors in cytoskeletal transport could impact RNA localized translation eliciting synaptic dysfunction and cognitive impairment.

To better understand questions pertaining to synapse biology and integrity, techniques have been optimized to collect synapses in the form of synaptosomes [29,30]. Synaptosomes are particles of synaptic material that form from the sheering forces during brain tissue homogenization. During homogenization, the dense, protein-rich membranes of synapses remain intact and pinch off from the larger plasma membrane allowing synapses to reseal and form individual particles which can be purified via gradient ultracentrifugation. The synaptosome model has frequently been used to analyze synaptic protein changes in AD [31–35]. This has led to discoveries including an upregulation of immune response pathways and downregulation of synaptic-signaling pathways [35]. Microarray analysis on AD human patient synaptosomes previously demonstrated an increase in localized synaptic genes and a decrease in metabolic genes. However, this data was not compared against expression changes in a brain homogenate background, and the analysis was limited to known mRNAs, precluding discovery and analysis of novel RNA species [36]. A study on mouse hippocampal synaptosomes derived from the 5xFAD model (bearing pathogenic variants in *APP* and *PSEN1*) that were droplet sequenced and compared to single nuclei sequencing showed synaptic mRNA localization differences in inflammatory pathway related genes [37]. Thus, synaptosomes offer an unparalleled opportunity to investigate the localized regulatory processes and mRNA expression differences in the context of brain aging and AD.

It is well-appreciated that several species of non-coding RNAs exist that fine-tune RNA and protein expression, including locally at the synapse. Circular RNAs (circRNAs) are non-coding RNAs serving regulatory functions that have been gaining more appreciation in the study of AD. CircRNAs are produced by back-splicing a downstream exon’s 3′ end, the “Donor”, to a preceding exon’s 5′ end, the “Acceptor”, creating a closed-loop structure. CircRNAs are primarily formed from conserved exon junctions, are stable due to exonuclease resistance, and can exceed linear counterparts in abundance by an order of magnitude [38–41]. Several properties of circRNAs lend themselves as unique regulators in neurobiology. Some circRNAs are translated and others can act as sponges for microRNAs [42–44]. Their expression is highest in the brain, especially synaptic vesicles and exosomes and accumulate during rodent postnatal development [45,46]. However, the role of circRNAs in synapses and in neurodegeneration and AD remains poorly understood.

To fill this gap, we herein utilized brain tissue from human AD samples and a transgenic mouse model of fAD to identify mislocalized coding and non-coding RNAs in synaptosomes. We discovered significant mRNA localization differences in AD brains compared to cognitively healthy controls. We also identified major differences among circRNAs localized to synapses in AD. Among our data, we discovered two unique isoforms of *circGSK3β* that are differentially expressed in AD. These differential expression changes among non-coding RNAs may reveal novel mechanisms of synaptic regulation critical to the development of AD and neurodegeneration.

## Results

### Synaptosome sequencing identifies mRNAs enriched and localized to synapses

We obtained post-mortem human brain samples from participants in the University of Washington (UW) Alzheimer’s Disease Research Center (ADRC) and the Adult Changes in Thought (ACT) Study based at Kaiser Permanente Washington [47]. We acquired frontal lobe (prefrontal cortex) samples from 23 individuals including donors with late-onset sAD and healthy controls. For 16 of these individuals, we also studied tissue samples from the temporal lobe (middle temporal gyrus). Patient samples were carefully selected to match for age, postmortem interval, and neuropathological features. A benefit from the ADRC and ACT programs is that the post-mortem interval (PMI) for collection is less than eight hours substantially reducing the impact of PMI as a confounding variable (**Tables 1 and S1**).

**Table 1:** Human brain sample properties. Brain tissue donors including sAD patients and cognitively healthy controls were recruited by the University of Washington Alzheimer’s Disease Research Center and the Kaiser Permanente Adult Changes in Thought (ACT) study. Brain tissue was collected from the frontal lobe (dorsolateral prefrontal cortex) of 23 individuals and temporal lobe (middle temporal gyrus) from 16 of the aforementioned 23 individuals. PMI: Postmortem Interval.

Group	Samples (n)	PMI (hrs)	Age (yrs)	Sex	Thal Phase	Braak Stage
**Frontal Lobe:**						
Control	12	4.8 ± 1.8	87.6 ± 6.7	11F, 1M	0–3	I—III
Sporadic AD	11	5.4 ± 1.4	86.0 ± 9.0	10F, 1M	4–5	V—VI
**Temporal Lobe:**						
Control	8	4.3 ± 0.8	87.3 ± 7.7	7F, 1M	0–3	I—III
Sporadic AD	8	5.6 ± 1.0	86.5 ± 4.1	7F, 1M	4–5	V—VI

**Fig 1:**
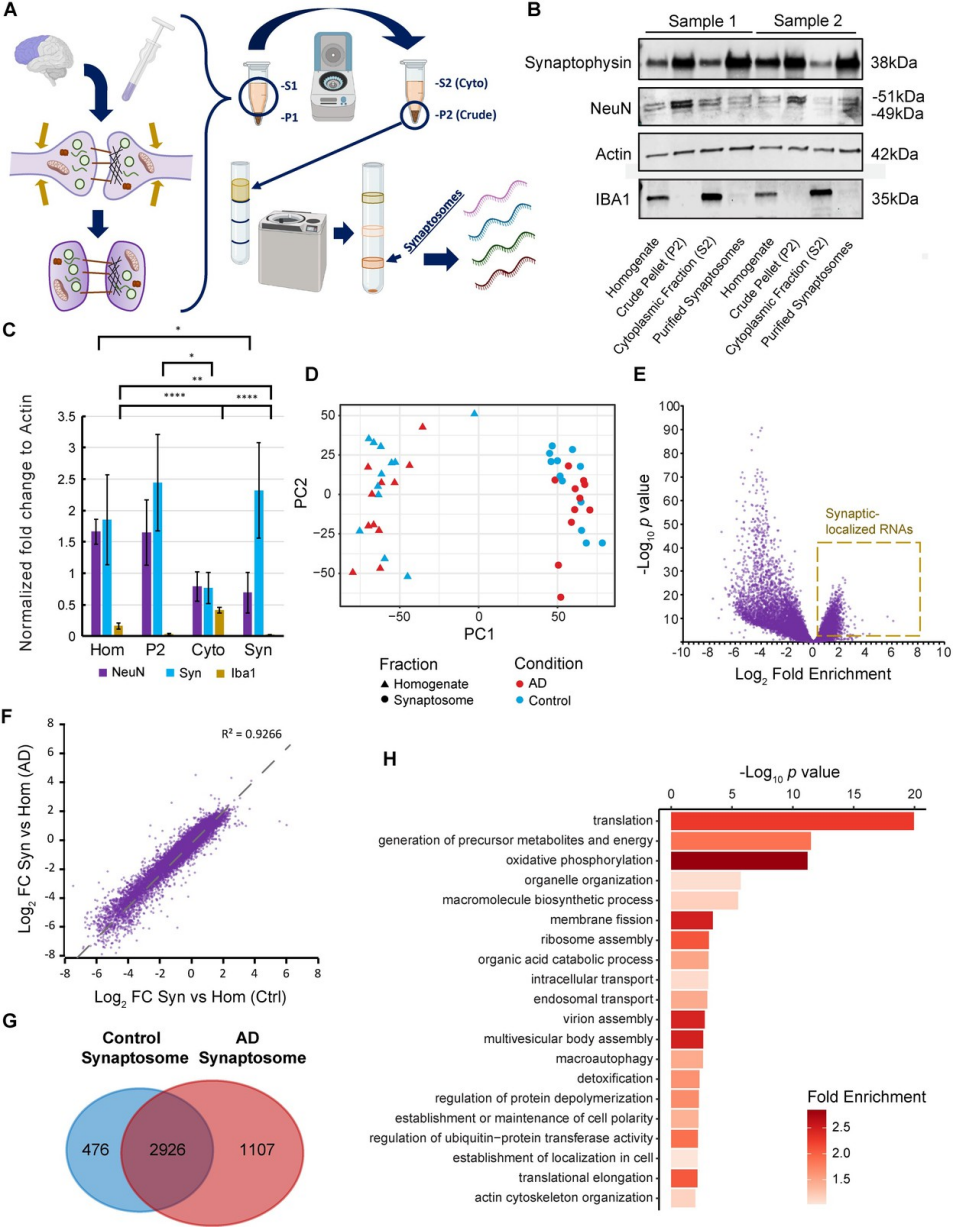
RNA sequencing of synaptosomes is an effective strategy for identifying localized RNA transcripts in the human frontal lobe. **A.** Schematic of synaptosome isolation, which are particles formed during the shearing forces of brain tissue homogenization whereby synaptic terminals pinch-off and reseal to form intact bipartite particles consisting of pre- and post-synaptic compartments. Based on their density, synaptosomes can be purified by sequential centrifugation steps concluding with gradient ultracentrifugation. After purification, the RNA contents of synaptosomes can be isolated and sequenced. *Figure created using a biorender*.*com license*. **B.** Western blot of a synaptic marker, synaptophysin, a nuclear marker, NeuN, and a microglial marker, IBA1, with Actin as a loading control for the different fractionation steps during synaptosome preparation in control samples. **C.** Quantification of the blot in (B). N = 4 per group. Significance calculated via T-test. *: p < 0.05, **: p < 0.01, ****: p < 0.0001. **D.** Multidimensional scaling analysis demonstrating separation between mRNA counts of homogenate and synaptosome fractions as well as variability between sporadic AD samples and controls. **E.** Volcano plot comparing synaptosome vs homogenate mRNA enrichment in only control samples. A distinct minority of mRNAs are significantly enriched at synaptic terminals (*p* < 0.01, gold box). **F.** Correlation between the fold change/enrichment of synaptosome vs homogenate between AD and control (R^2^ = 0.9266). **G.** Venn diagram demonstrating overlap of synaptosome enriched genes between AD and control samples. **H.** GO biological process analysis of synaptosome-enriched genes.

We isolated synaptosomes from frontal lobe brain samples using a well-established sucrose gradient protocol (**Fig 1A**) [29,30]. We validated our technique by western blot of common markers demonstrating enrichment of synaptophysin in the synaptosome fraction and depletion of the neuronal marker NeuN as well as the robust elimination of the glial marker IBA1 in control samples (**Fig 1B** and **1C**). We saw similar levels of TDP-43, SNAP25, COX4, and RAB4 among all fractions (**S1A** and **S1B Fig**). Fractionation was similar in the AD samples (**S1C Fig**). Furthermore, for a visual confirmation, we imaged particles in the synaptosome fraction using a fluorescent lipophilic stain by confocal microscopy. We observe particles of the expected .5–1.5μm size, several of which appear as bipartite structures indicative of joined pre-synaptic and post-synaptic terminals (**S1D Fig**) [48]. After synaptosome isolation, we extracted RNA from the bulk synaptosome fraction as well as from the pre-fractionated homogenate that served as a background with which to compare the expression at synapses. We subsequently performed RNA sequencing using a ribo-removal stranded total RNA kit to capture coding RNAs and non-coding RNAs lacking poly-A tails, such as circRNAs. After measuring read counts, we analyzed sample variability using sample distance clustering and multidimensional scaling analysis (**Figs 1D** and **S1E**). This analysis revealed robust clustering distinctly separating synaptosome and homogenate fractions as well as between AD and control albeit with more variability among the AD samples compared to the control samples. We ran hierarchal clustering on the expression data and discovered no other confounding variables except a possible contribution from patient age (**S1F Fig**). Critically, we did not identify PMI, RNA integrity number (RIN), sex, or *APOE* alleles, to be confounding variables. The lack of any other variables confounding our analyses of interest on Fraction, and Condition after batch correction was confirmed by weighted gene correlation analysis (see methods and **S17 Fig**). We repeated multidimensional scaling analysis focusing on groups of samples including only control (**S2A Fig**) and only AD (**S2B Fig**) which substantiated robust separation of homogenate vs. synaptosome in both groups. We also looked at only homogenate (**S2C Fig**) and only synaptosome (**S2D Fig**) which showed more distinct profiles among AD vs control in the synaptosome fraction compared to the homogenate.

To identify RNAs enriched at synapses in the frontal lobe, we first compared RNA abundance differences between synaptosomes versus the pre-fractionated homogenate in control samples. This resulted in 3,402 significantly enriched mRNAs at synapses (p < 0.01). Notably, synaptically-enriched mRNAs are still a minority of all measured mRNAs detected in control tissue while most mRNAs were still preferentially localized elsewhere in the bulk homogenate including in the cytoplasm, nucleus, and non-neuronal cells (**Fig 1E** and **S2 Table**). We similarly analyzed the synaptosome transcriptome in the frontal lobe AD samples (**S1G Fig** and **S3 Table**). We observed a very strong correlation between the control and AD samples (R^2^ = 0.9266) (**Fig 1F** and **1G**). Successful synaptic-transcriptome enrichment was validated by high expression of PSD-95 (Discs Large MAGUK scaffold protein 4; *DLG4*) in synaptosomes vs. homogenate. Other notable synaptic transcripts include: activity related cytoskeleton associated protein (*ARC*), calcium/calmodulin dependent protein kinase II alpha (*CAMK2A*), and SH3 and multiple ankyrin repeat domains 3 (*SHANK3*). Another telltale signature of synaptosome fractionation is the alternative splicing of fused in sarcoma (*FUS*) mRNA where we observe intron retention isoforms specifically in nuclear transcripts (**S3 Fig**) [49]. We also analyzed GO biological process enrichment in the synaptic transcriptome and discovered that synaptic-localized mRNAs are significantly enriched in translation, metabolic functions, and organelle organization processes (**Fig 1H**). To this end, some notable gene families are represented among our synapse-enrichment data including several eukaryotic translation initiation factors (*EIF1-EIF6*). We also observed an enrichment of motor proteins including dyneins and kinesins, as well as RNA-binding proteins including Fragile-X mental retardation protein (*FMR1*).

### Significant mRNA localization differences occur at AD synapses

**Fig 2:**
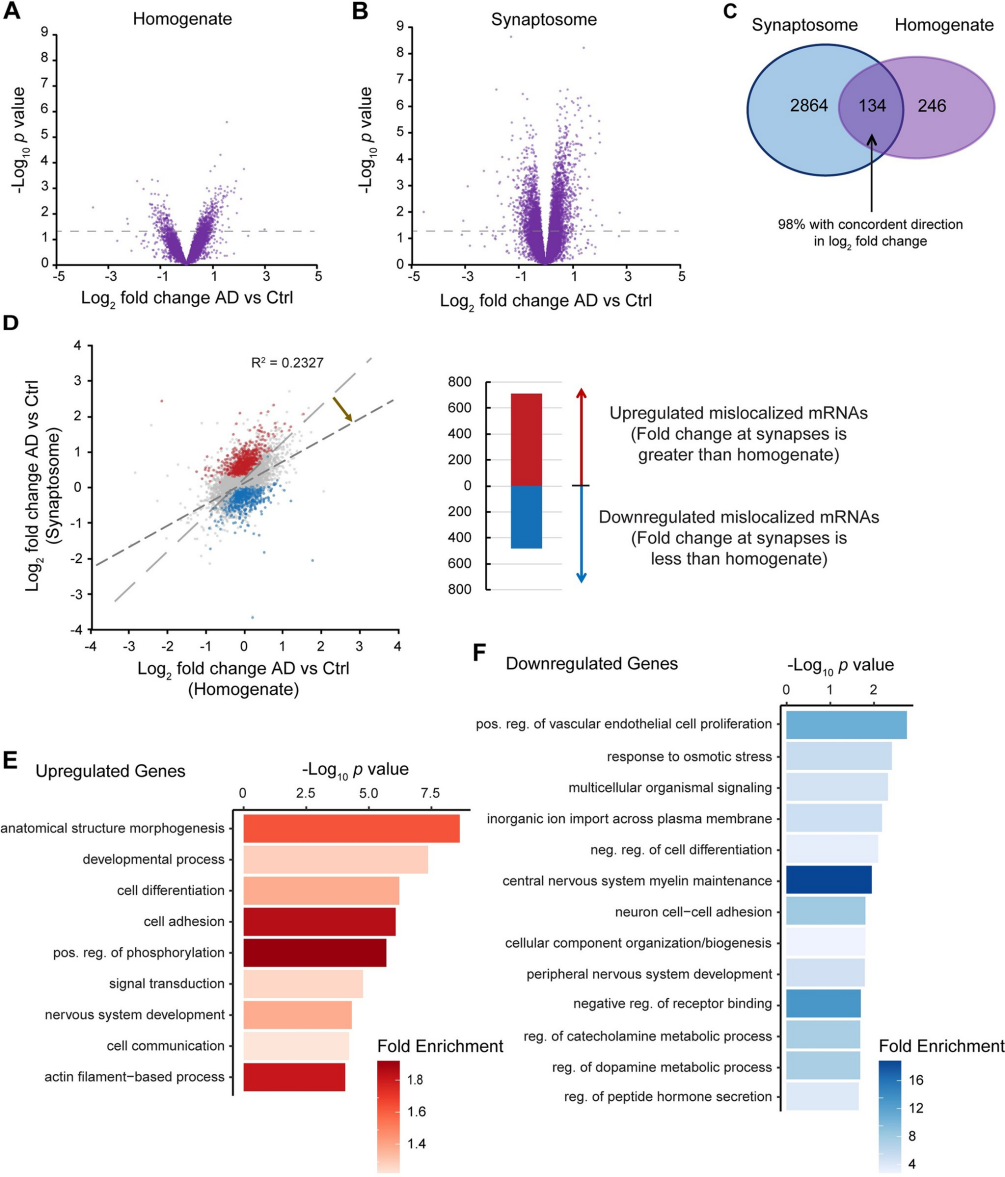
Comparison between AD and control in synaptosomes vs homogenate reveals mislocalized transcripts in the human frontal lobe. **A.** Volcano plot comparing expression differences between AD and control in the bulk, unfractionated homogenate. **B.** Volcano plot comparing expression differences between AD and control just within the synaptosome particles. **C.** Venn diagram comparing the significant expression differences in homogenate (**A**) and synaptosome (**B**). **D.** Comparison of the fold changes of AD vs control between synaptosomes and homogenate (R^2^ = 0.2327). Genes that have a T-statistic most distant from a y = x trendline are significantly mislocalized, independent of global expression changes (FDR < 0.001, see methods). **E.** GO biological process analysis of upregulated genes in (**D**). **F.** GO biological process analysis of downregulated genes in (**D**).

First, we analyzed the frontal lobe sequencing data from synaptosomes and homogenate separately and compared AD and control in each case (**Fig 2A** and **2B**; **S4** and **S5 Tables**). We saw substantially more differentially expressed mRNAs (2,998; *p* < 0.05) in the synaptosome data, compared to the homogenate (380; *p* < 0.05). 134 of these mRNAs overlapped and 98% were concordant in their direction of log_2_ fold-change expression differences (**Fig 2C**). Given the high concordance between synaptosome and homogenate fractions, it may be the case that significantly differentially expressed mRNAs at synapses are merely a result of global expression changes. Therefore, we wanted to understand the extent to which mRNA differences at the synapse in AD occurred as a result of changes in how mRNAs were transported and localized to the synapse in addition to global expression changes. We refer to this as “mislocalization” in which the expected fraction of a transcript’s reads sequenced within the synaptosome fraction compared to the homogenate is different in AD versus control. The exact mechanism by which an mRNA becomes mislocalized in AD has yet to be determined, though it could be hypothesized this is a result of errors in cytoskeletal transport or the degradation dynamics of mRNAs within neurites.

To identify mislocalized mRNAs at synapses in AD, we compared the log_2_ fold change between AD and control of each mRNA in synaptosomes to that of AD vs control in homogenate (**Fig 2D**). This analysis showed that mRNA expression differences between AD and control in synaptosomes are largely uncorrelated with global expression changes and deviates from a 1:1 trendline (R^2^ = 0.2327). To identify mislocalized RNAs, we calculated the ratio of log_2_ fold change of AD to control in the synapse to the log_2_ fold change of AD to control in the homogenate to determine the fold change of mislocalization. We calculated a *p* value based on the deviation from a 1:1 (i.e. y = x) trendline by assuming a normal distribution while also accounting for the standard error of the respective log_2_ fold change measurements (see methods for additional details). This analysis resulted in the identification of 1,195 significantly mislocalized mRNAs in the frontal lobe synapses of AD brains (FDR < 0.001), 712 of which were upregulated and 483 were downregulated (**S6 Table**). We found that by clustering results and visualizing them in a heatmap, these mislocalized mRNAs form distinct blocks representing independent expression changes in synaptosomes versus homogenate (**S4A Fig**). Gene ontology analysis of the upregulated mRNAs revealed enrichment of anatomical structure morphogenesis, developmental process, cell differentiation, and cell-adhesion biological processes (**Fig 2E**). The downregulated mRNAs showed enrichment of vascular endothelial cell proliferation, response to osmotic stress, signaling, and ion import across the plasma membrane (**Fig 2F**).

**Fig 3:**
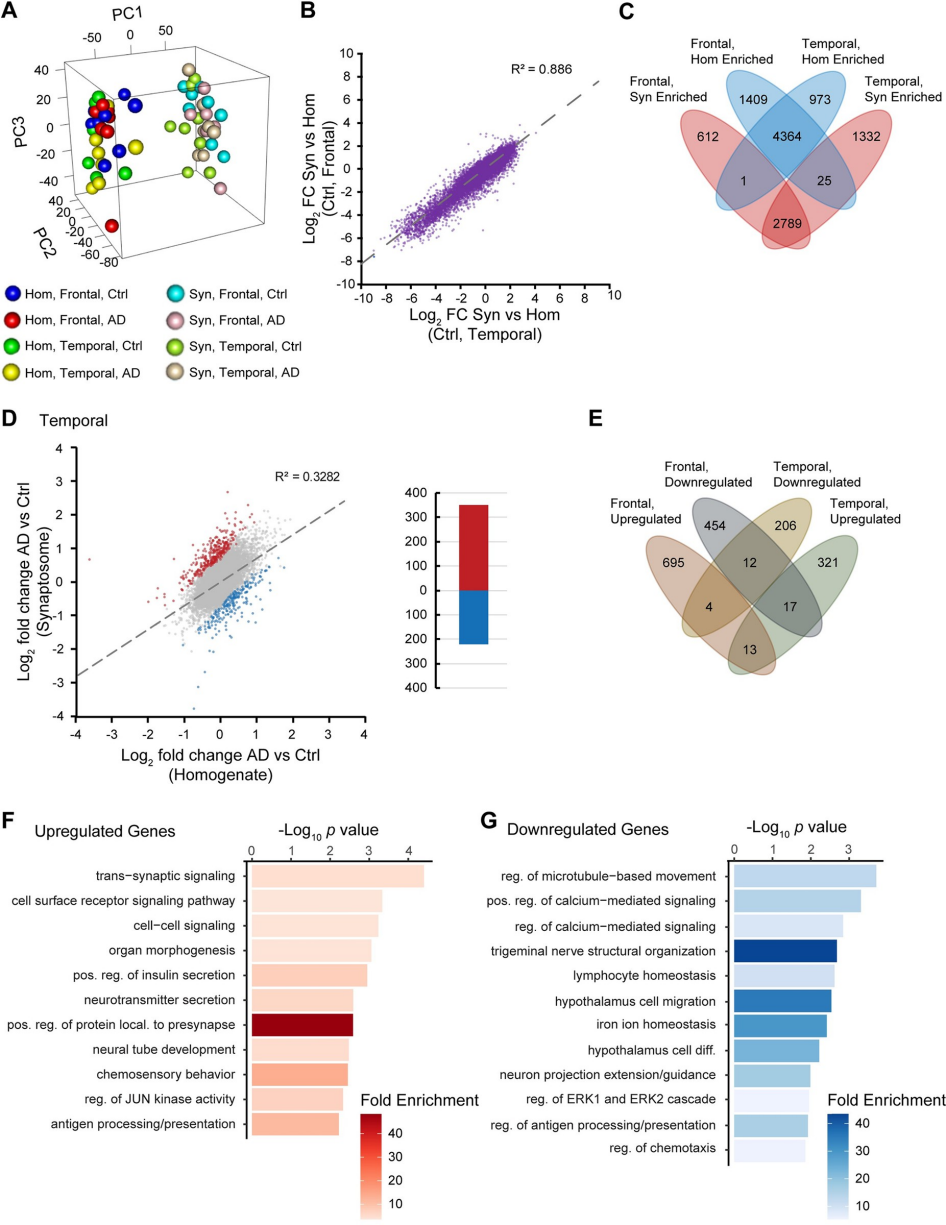
Synaptosome sequencing in the human temporal lobe reveals similarities among synaptosome-enriched transcripts and differences in mislocalized transcripts. **A.** Multidimensional scaling analysis among the frontal lobe and temporal lobe samples. **B.** Comparison of synaptosome-enriched transcripts between frontal and temporal lobe samples (R^2^ = 0.886). **C.** Venn diagram showing overlap of synaptosome-enriched transcripts between frontal and temporal lobe samples (*p* < 0.01). **D.** Comparison of the fold changes of AD vs control between synaptosomes and homogenate in temporal lobe samples (R^2^ = 0.3282). Genes that have a T-statistic most distant from a y = x trendline are significantly mislocalized, independent of global expression changes (FDR < 0.01, see methods). **E.** Venn diagram showing minimal overlap of mislocalized transcripts between frontal and temporal lobe samples. **F.** GO biological process analysis of upregulated genes in (**D**). **G.** GO biological process analysis of downregulated genes in (**D**).

Next, we compared results from frontal lobe to results from the temporal lobe. First, we ran sample distance clustering and multidimensional scaling analysis. Similar to the frontal lobe, we observed robust separation between the synaptosome and homogenate fractions as well as between AD and control (**S5A** and **S5B Fig**). We then ran multidimensional scaling analysis in 3D to compare the frontal lobe samples to the temporal lobe samples (**Fig 3A**). This analysis revealed that principal component 1 distinctly separated the synaptosome and homogenate fractions among both frontal lobe and temporal lobe samples, principal component 2 appeared to explain variation between AD and control, while principal component 3 appeared to distinguish between the frontal lobe and temporal lobe brain regions, notably with less variation that that of the other components. Sample distance clustering similarly reflected these observations (**S5C Fig**). Via heatmap, it appeared that clustering of disease status defined sample mRNA expression patterns preceding the clustering of brain regions, and most importantly synaptosome versus homogenate took overall precedence (**S6A Fig**).

Next, we analyzed synaptic enrichment of mRNAs in the temporal lobe. There was a similar pattern where a minority of mRNAs were synaptosome enriched (**S7A Fig and S7 Table**). This was also observed in the AD temporal lobe tissue with a strong correlation between AD and control (R^2^ = 0.8955) (**S7B** and **S7C Fig and S8 Table**). Comparing synaptic enrichment between temporal lobe and frontal lobe among our control samples also showed a strong correlation (R^2^ = 0.886) (**Fig 3B** and **3C**). We analyzed AD versus control in the temporal lobe separately between the homogenate and synaptosome fractions and discovered many more significant differences in mRNA expression between them (*p* < 0.05). 568 of these differentially expressed mRNAs overlapped between the fractions with 99% having a concordant directionality in log_2_ fold change (**S7D–S7F Fig; S9** and **S10 Tables**).

We repeated our mislocalization analysis on the temporal lobe samples. We found a moderately stronger (41% larger) correlation between synaptosome and homogenate expression differences between AD and control (R^2^ = 0.3281) (**Fig 3D**). Given a stronger correlation, there were fewer mislocalized genes in the temporal lobe versus the frontal lobe with very little overlap between the two brain regions (**Fig 3E**). This showed only 351 upregulated mRNAs and 222 downregulated ones (FDR < 0.01; **S11 Table**). GO analysis indicated enrichment of trans-synaptic signaling, cell surface receptor signaling, neurotransmitter secretion, and protein localization to the presynapse biological processes among upregulated mRNAs (**Fig 3F**). Among downregulated mRNAs there was an enrichment of microtubule-based movement regulation, regulation of calcium-mediated signaling, and neuron projection guidance (**Fig 3G**). As was the case for the frontal lobe, hierarchical clustering of mRNA expression showed distinct blocks representing disproportional expression changes between AD and control between the synaptosome and homogenate fractions (**S7G Fig**).

### Synaptosome sequencing findings in the APP/PSEN1 mouse model are poorly correlated with findings from human samples

Finally, given the difficulties with studying functional consequences of mislocalized RNAs in human samples, we wanted to examine whether the well-studied APP^swe^/PSEN1^dE9^ mouse model recapitulated the differences we observed in our human datasets[50]. We sequenced homogenates and synaptosome fractions from mouse frontal cortex tissue including six WT control and eight APP/PSEN1 aged mutant mice matched closely for age (**S8A Fig**). Using sample distance clustering and multidimensional scaling analysis, we found more robust separation and less variation between AD and WT compared to the human samples in addition to the distinct separation between the synaptosome and homogenate fractions (**S8B** and **S8C Fig**).

**Fig 4:**
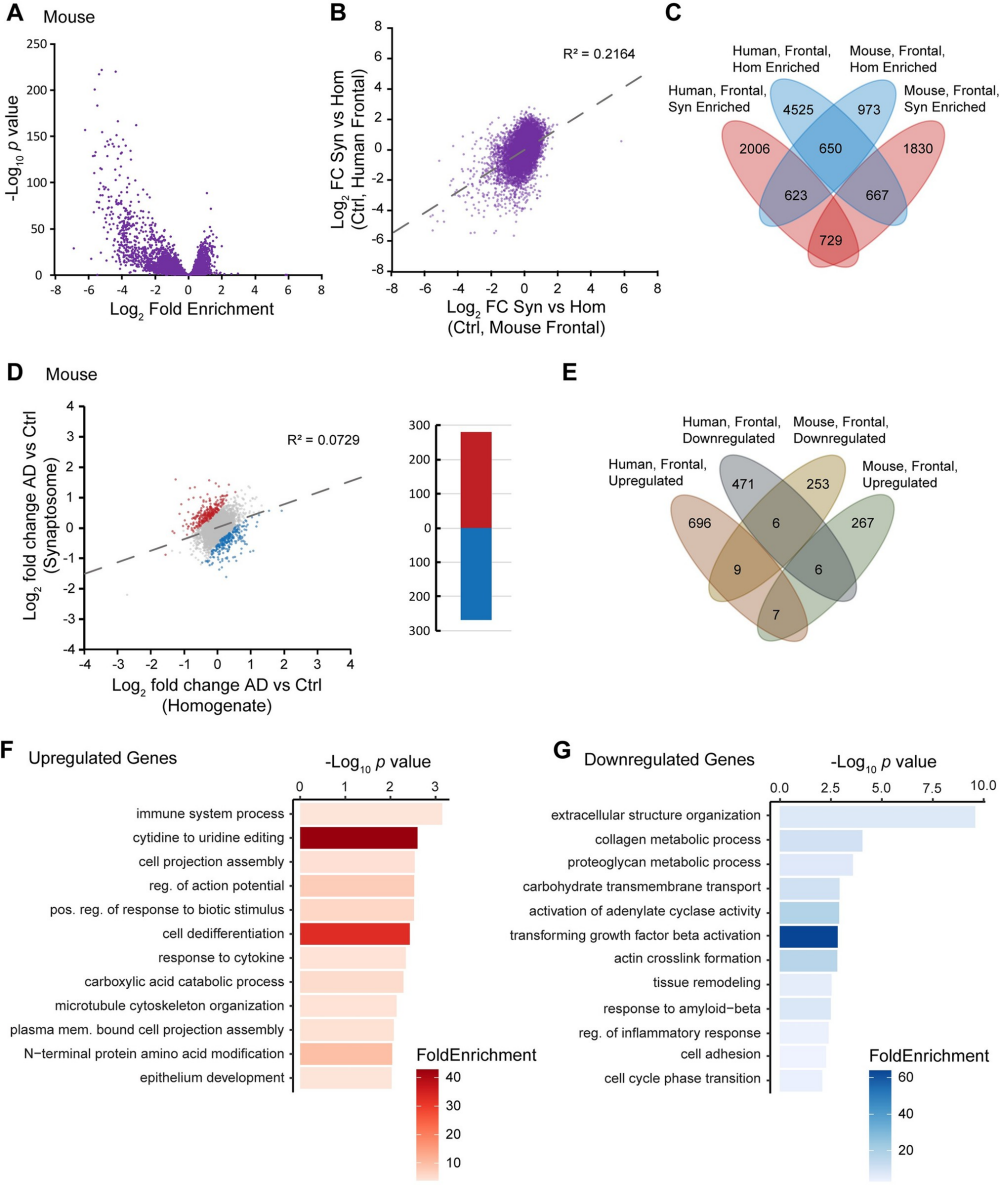
Synaptosome sequencing of frontal lobe samples from the APP^swe^/PSEN1^dE9^ AD and wild-type mouse models reveals substantial differences in transcript localization patterns from that of human samples. **A.** Volcano plot comparing synaptosome vs homogenate in wild-type mouse samples (FDR < 0.001). **B.** Comparison of synaptosome-enriched transcripts between mouse and human frontal lobe samples (R^2^ = 0.2164). **C.** Venn diagram showing overlap of synaptosome-enriched transcripts between human and mouse frontal lobe samples (*p* < 0.01). **D.** Comparison of the fold changes of AD vs control between synaptosomes and homogenate in temporal lobe samples (R^2^ = 0.0729). Genes that have a T-statistic most distant from a y = x trendline are significantly mislocalized, independent of global expression changes (FDR < 0.01, see methods). **E.** Venn diagram showing minimal overlap of mislocalized transcripts between mouse and human and mouse frontal lobe samples. **F.** GO biological process analysis of upregulated genes in (**D**). **G.** GO biological process analysis of downregulated genes in (**D**).

When we analyzed the sequencing data between synaptosome and homogenate in mouse frontal lobe, we also found a small selection of mRNAs that were synaptic enriched, which was also strongly correlated between AD and control (R^2^ = 0.8913) (**Figs 4A**, **S9A**, and **S9B**; **S12** and **S13 Tables**). However, we did not find this model to reflect the differences we observed among our human frontal lobe samples. Comparing synaptic enriched mRNAs between control mouse and human frontal lobe tissue, we observed a weak correlation (R^2^ = 0.2164) with very little overlap between the two species (**Fig 4B** and **4C**). Next, we looked at significant expression differences between AD and control separately in homogenate and synaptosome. We found that 105 mRNAs overlapped between the fractions with 92% having concordant directionality in log_2_ fold change (**S9C–S9E Fig**; **S14** and **S15 Tables**).

We conducted our mislocalization analysis and found 280 upregulated and 268 downregulated mRNAs. Only 13 of these mRNAs overlapped with our human frontal lobe data (**Fig 4D** and **4E**; **S16 Table**). GO analyses seemed to contrast with the human data as well with upregulated mRNAs being enriched for immune-related biological processes and cell projection assembly (**Fig 4F**). In addition, downregulated mRNAs were enriched for extracellular structure organization, carbohydrate transmembrane transport, transforming growth factor beta activation, and (interestingly) response to amyloid-beta (**Fig 4G**).

### Our mouse synaptosome data aligns with independent studies

In 2023, a study was published in which individual 5xFAD and control mouse hippocampus synaptosomes and nuclei were sorted into droplets and sequenced [37], though concerns have been raised regarding the study’s methodology and synaptosome purity [51]. We conducted a comparison analysis between our data and Niu et al’s to highlight differences in our approaches.

There are notable differences between our methods presented here and Niu et al.’s approach [37]. The most significant is that instead of using bulk pre-fractionated homogenate as the background, Niu et al. used single nuclei RNAseq as their background to compare synaptosome localization. The drawback is that this approach would only identify nascent mRNAs while the benefit being that cell type diversity, specificity, and clustering can add new dimensions to interpreting the data. Remarkably we found substantial similarity in synaptic-localized mRNAs with a modest correlation of R^2^ = 0.58 and an overlap of 1155 genes (**S10A** and **S10B Fig**) while a minority of mRNAs show opposite compartmental enrichment.

We repeated our mislocalization analysis on their data and discovered an entirely different repertoire of mislocalized mRNAs within the mouse hippocampus reflecting tissue differences akin to the differences observed between human frontal and temporal lobes as well as differences between the 5xFAD and APP/PSEN1 mouse models (**S10C Fig**). We found that 147 genes were upregulated including *Nrxn1/3*, *Calm3*, and *Camk2a/b*, as well as 21 downregulated including *Mef2c* and *Septin7*.

### Mislocalized mRNAs are enriched for particular microRNA (miRNA) binding sites

A 2022 publication presented a study in which miRNA abundance was quantified in AD and control human synaptosomes which found an upregulation of miR-502-3p, miR-103a-3p, and miR-501-3p among AD synaptosomes [52]. In an attempt to postulate mechanisms relating to mislocalized mRNAs, we analyzed miRNA binding sites in the 3′UTRs of differentially expressed mRNAs and found striking patterns. Among the frontal cortex upregulated mislocalized mRNAs, we observed an enrichment of 241 miRNA binding sites compared to background frequencies (FDR < 0.05) including miR-101, miR-203, and miR-501-5p (**S11A Fig** and **S17 Table**). We found 309 binding sites that were enriched among the frontal lobe downregulated mRNAs including miR-10, miR-22, miR-138, and miR-503 (**S11B Fig** and **S18 Table**). A notable observation is that miRNAs that are enriched among the upregulated mRNAs are correspondingly de-enriched among the downregulated mRNAs and vice versa. In the temporal upregulated mislocalized mRNAs, we saw an enrichment of 360 sites including miR-22, miR-138, miR-151, and miR-503 (**S11C Fig** and **S19 Table**). In the temporal downregulated mRNAs, we saw an enrichment of only 8 miRNA binding sites including miR-296-3p, miR-145 and miR-181 (**S11D Fig** and **S20 Table**). These data can support the hypothesis that these miRNAs are also mis-expressed in AD and contribute towards mRNA stability at synapses suggesting a mechanism behind their mislocalization.

### CircRNAs are enriched at synapses and show significant localization differences in AD

**Fig 5:**
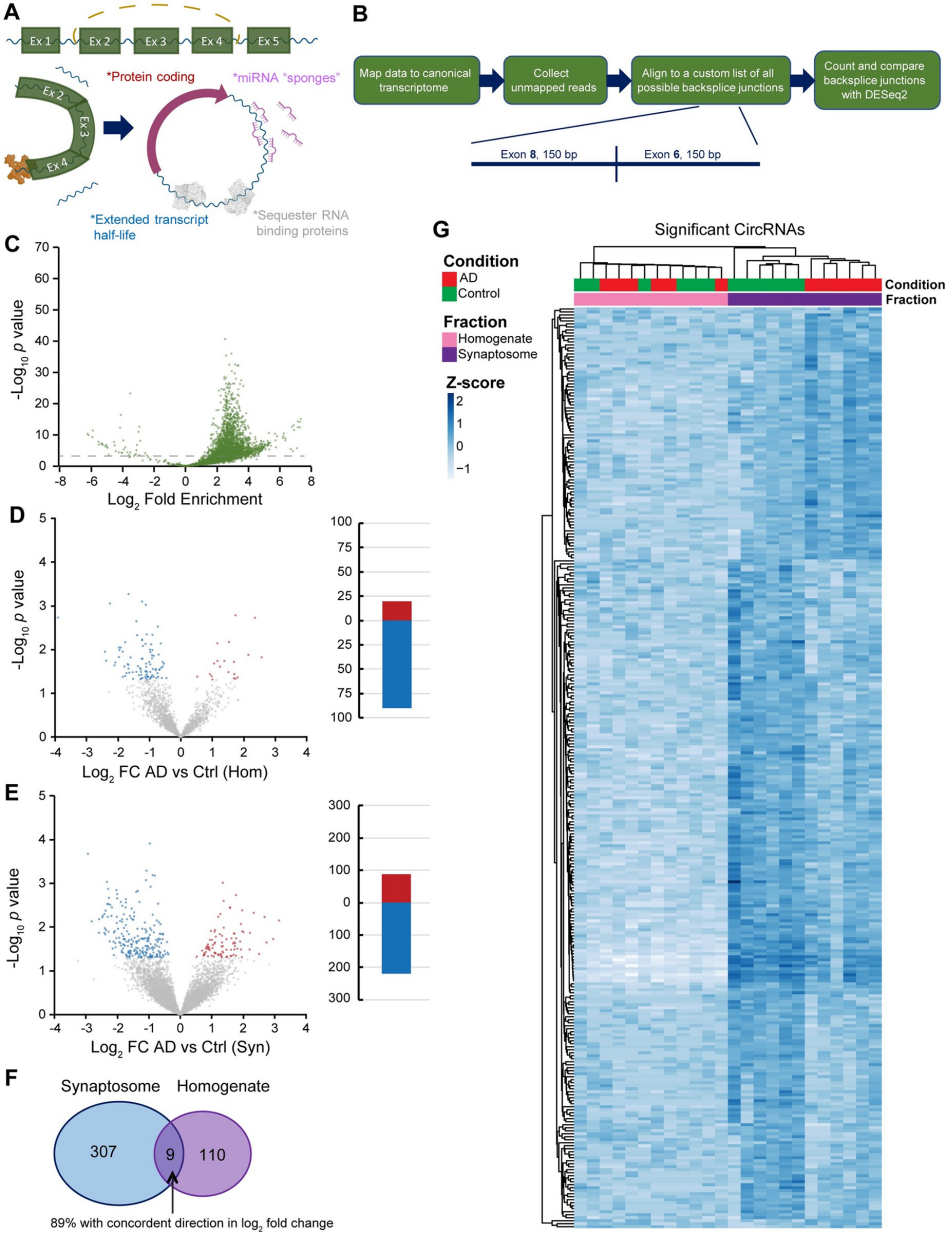
Analysis of circular RNAs in synaptosomes shows their preferential localization to synapses and substantial differences in AD samples. **A.** Schematic of circular RNAs (circRNAs) that are formed by back-splicing a later exon onto a preceding exon. *Figure created using a biorender*.*com license*. **B.** Workflow for analyzing circRNAs includes first mapping the sequencing data to the canonical transcriptome. Second, unmapped reads are extracted from the alignment file. Third, unmapped reads are aligned to a custom list of all possible back-splice junctions within genes including 150bp flanking each side. Finally, reads mapping to back splice junctions are counted and differential expression is evaluated using DESeq2. **C.** Volcano plot comparing circRNAs in synaptosome vs homogenate in control frontal lobe samples. The overwhelming majority of circRNAs are significantly enriched at synaptic terminals. **D.** Volcano plot comparing circRNAs in AD vs control in the unfractionated homogenate (*p* < 0.05). **E.** Volcano plot comparing circRNAs in AD vs control in the synaptosome fraction (*p* < 0.05). **F.** Venn diagram showing overlap of AD vs control differentially expressed circRNAs between the homogenate and synaptosome fractions. **G.** Heatmap of differentially expressed circRNAs between AD vs control in synaptosomes. Differential expression is both independent of, and substantially greater than, the bulk homogenate.

Despite considerable advances in circRNA biology, the primary mechanistic role(s) of most circRNAs are still uncertain (**Fig 5A**). We wanted to understand whether there are differences among circRNA species localized to synapses in AD. To identify circRNAs, we analyzed our sequencing data for reads mapping to back-splice junctions by an alignment strategy where we first mapped our RNA sequence reads to canonical transcripts and subsequently extracted the unmapped reads. From there, we aligned the reads to a file containing all possible back splice junctions including 150 bp on either side within each canonical transcript (**Fig 5B**).

First, we calculated expression differences between synaptosomes and pre-fractionated homogenate from human frontal lobe control samples (**Fig 5C** and **S21 Table**). We found that the overwhelming majority (2,276) of circRNAs are enriched and localized to synapses compared to unfractionated homogenate (*p* < 0.001). An identical pattern was seen in the frontal lobe AD samples, and correlation between AD and control was moderate (R^2^ = 0.3928) (**S12A** and **S12B Fig** and **S22 Table**). This is consistent with observations from previous studies in rodents demonstrating that circRNAs are enriched at synapses [39,46,53,54]. Next, we compared circRNA expression differences between AD and control in the frontal lobe homogenate samples (**Fig 5D** and **S23 Table**). Here we found 20 upregulated and 90 downregulated circRNAs (*p* < 0.05). Given the observation of high synaptic enrichment of circRNAs, we focused on differences between AD and control among the synaptosome data. We found more substantial differences in circRNA synaptic expression including 87 upregulated and 220 downregulated circRNAs (**Fig 5E** and **S24 Table**). Only 9 of these differentially expressed circRNAs overlapped between the homogenate and synaptosome fractions (**Fig 5F**). We visualized these circRNA expression trends by hierarchical clustering (**Fig 5G**). From this we confirmed that expression changes in circRNAs between AD and control take place exclusively at the synapse independent of changes in the unfractionated homogenate. To visualize how circRNA expression varies across the samples, we conducted multidimensional scaling analysis exclusive to circRNA counts (no linear counts). We observed more variation among the synaptosome samples between AD and control, and more uniformity among the homogenate samples (**S12C Fig**). Another intriguing observation was the predicted length of circRNAs we observed. The majority of circRNAs have back-splice junctions that are between 2 and 4 exons apart (**S12D Fig**). Furthermore, a number circRNAs consist of only a single exon spliced back on itself.

One of the most noteworthy discoveries from the frontal lobe synaptosome circRNA data was the observation of isoform switches within the same genes between AD and control (**Table 2**). For example, *ATF6* showed an exon 9-to-2 isoform that is downregulated in AD synapses while simultaneously there is an exon 15-to-10 isoform that was upregulated.

**Table 2:** Examples of circRNA isoform switches between sAD and control in frontal lobe synaptosomes. Comparing RNA sequencing data mapped to circRNA back-splice junctions between AD and control synaptosomes revealed that significant expression changes often arose from multiple circular isoforms originating from the same gene and would show contrasting fold changes. “Donor” refers to the downstream exon by which its 3′ end is spliced back onto the upstream “Acceptor” exon’s 5′ end. We report the raw *p* value.

Gene	Donor	Acceptor	Log_2_ Fold Change	*p* value
*ATF6*	9	2	-2.81	0.00740
*ATF6*	15	10	1.94	0.00860
*ATF6*	14	10	-1.25	0.0363
*ANKS1B*	4	2	1.21	0.000585
*ANKS1B*	8	2	-1.01	0.0170
*GSK3β*	9	7	1.58	0.00370
*GSK3β*	10	9	-1.68	0.00666
*UBA2*	16	9	-1.84	0.00755
*UBA2*	16	10	1.49	0.0473

When analyzing the human temporal lobe data, there was a similar trend where circRNAs are substantially enriched in the synaptosome fraction (**S13A Fig** and **S25 Table**). We also observed a moderate correlation of circRNA synaptic enrichment between temporal and frontal lobes (R^2^ = 0.4843) (**S13B Fig**). We similarly conducted a multidimensional scaling analysis among the temporal lobe samples exclusive to circRNA counts. This showed similar trends to the frontal lobe circRNA clustering (**S13C Fig**). In the temporal lobe homogenate fraction, we observed 99 upregulated and 35 downregulated circRNAs in AD versus control (**S13D Fig** and **S26 Table**). In the temporal lobe synaptosome fraction, we found 218 upregulated and 52 downregulated circRNAs (**S13E Fig** and **S27 Table**). Between the temporal lobe synaptosome and homogenate fractions, only 10 of these circRNAs overlap as differentially expressed between AD and control (**S13F Fig**). Finally, between temporal lobe synaptosomes and frontal lobe synaptosomes, 17 circRNAs overlap with only 65% as being similarly differentially expressed between AD and control (**S13G Fig**).

CircRNAs among our mouse frontal lobe samples demonstrated little similarity to what we observed in the human samples. Though we found that circRNAs are still prominently enriched in synaptosomes, these identifications were less abundant and pronounced, encapsulating only 200 circRNAs (*p* < 0.01) (**S14A Fig** and **S28 Table**). Among mouse frontal lobe synaptosomes, between AD and control, we saw upregulation of 83 circRNAs and downregulation of 83 circRNAs (**S14B Fig** and **S29 Table**).

We sought to benchmark our novel approach to identifying circRNAs against another popular software, DCC [55] which has been used previously in AD research [56]. In contrast to the DCC method which relies on the chimeric junction files produced by RNA STAR, we create a custom fasta junction reference which substantially narrows the search space and boosts sensitivity to identify unique splice isoforms of coding exons (as described in **Fig 5B**). We tested DCC on our human frontal lobe RNA seq data following standard DCC workflow and settings, and the software detected 2374 total circRNAs, while our approach detected 3999 circRNAs. We repeated our synaptosome analysis using the DCC data and found 1081 circRNAs significantly enriched in synaptosomes, 752 of which aligned with our data (which discovered 2,276; **S30 Table**). Next, we compared AD synaptosomes to control synaptosomes and DCC found 296 significant differentially expressed circRNAs, 45 of these overlapped with our finding of 307 circRNAs (**S31 Table**).

### *CircGSK3β* isoforms show contrasting expression changes at AD synapses and can regulate protein and phosphorylation levels including for tau

**Fig 6:**
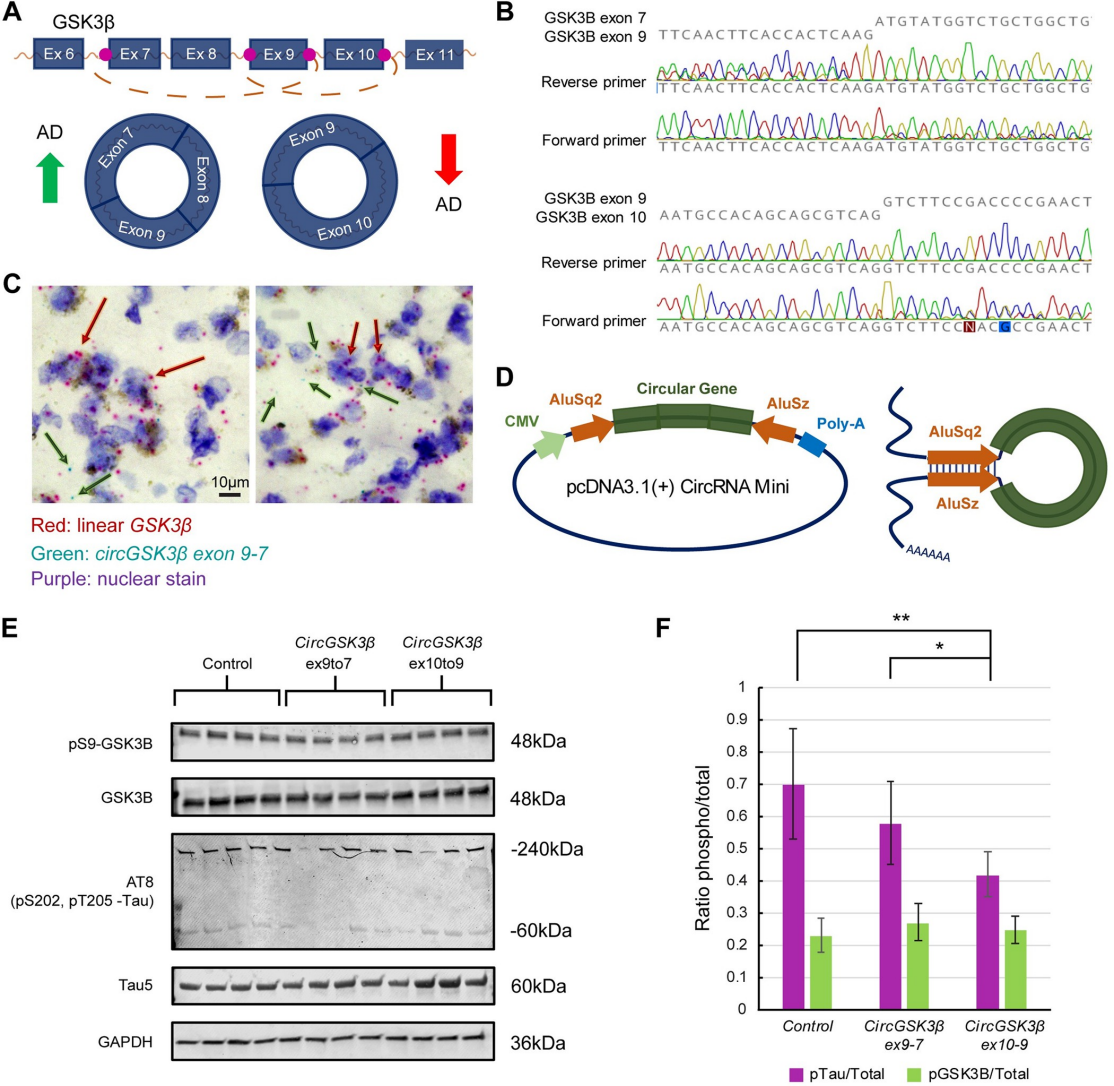
*CircGSK3β* isoforms show contrasting expression differences between AD and control and may play regulatory roles related to AD pathologies. **A.** Two distinct isoforms of *circGSK3β* with contrasting expression differences in AD. An isoform where exon 9 is back-spliced onto exon 7 is upregulated in AD, while an exon 10 spliced onto exon 9 isoform is downregulated. *Figure created with a biorender*.*com license*. **B.** Confirmation of *circGSK3β* isoform back-splice junctions by Sanger sequencing using primers flanking the splice junction. **C.** BaseScope RNA hybridization probes show that *circGSK3β* isoforms (green) are more frequently distal to the nucleus than the linear *GSK3β* transcript (red) in human control frontal lobe brain slices. Tissue and nuclei were stained with hematoxylin. **D.** A specialized plasmid containing Alu elements coerces circular back-splicing of gene insert [59]. **E.** Western blot of neuron-differentiated SH-Sy5y cells transfected with either a control plasmid, a *circGSK3β exon 9–7* plasmid, or a *circGSK3β exon 10–9* plasmid. Cell lysates were probed for total Tau (Tau5), phospho-Tau (AT8), GSK3β, phospho-S9 GSK3β, and GAPDH. **F.** Quantification of the ratios between phospho-Tau and total Tau as well as between phospho-S9 GSK3β and total GSK3β in each of the transfection conditions in (**E**). N = 8 samples per group. Significance calculated by T-test. *: *p* < 0.05, **: *p* < 0.01, ***: *p* < 0.001.

One of the most intriguing discoveries from our human data concerned two distinct isoforms of *GSK3β*. A *circGSK3β* exon 9-to-7 isoform was upregulated in AD with a log_2_ fold change of 1.58, while a *circGSK3β* exon 10-to-9 isoform was downregulated in AD with a log_2_ fold change of -1.68 (**Fig 6A**). Importantly, these isoforms have not been previously established nor associated with AD. The GSK3β protein plays a well-established role in AD pathology given it is among the chief proteins that phosphorylate tau [47,57,58]. We validated the back-splice junctions using PCR and Sanger sequencing with an exon 9 forward primer and an exon 7 reverse primer and an exon 10 forward primer and an exon 9 reverse primer respectively (**Fig 6B**), and used similar methods to confirm another significant circRNA present in *HOMER1* (**S15A Fig**). To validate synapse localization and sequencing quantitative measurements we probed human frontal cortex brain sections using BaseScope RNA *in situ* hybridization. We discovered that, indeed, these *circGSK3β* isoforms have a propensity to localize distal from the soma while the linear *GSK3β* isoform tends to be localized more proximal to the nucleus (**Figs 6C** and **S15B**). We confirmed distal enrichment by quantifying relative distance from the soma as marked by the nucleus (**S15C Fig**). We also discovered supporting evidence of AD versus control expression differences of *circGSK3β* isoforms by analyzing BaseScope images (**S15D Fig**).

To investigate the biomolecular impacts of the *circGSK3β* isoform expression, we utilized a plasmid vector that coerces circRNA splicing by inserting circRNA-producing exons between two Alu elements (**Fig 6D**) [59]. We used this vector to transfect neuron-differentiated SH-Sy5y cells with *circGSK3β* isoforms and subsequently measured protein expression. We used a differentiation protocol incorporating retinoic acid and BDNF. We confirmed neural morphology as visualized under a light microscope (**S15E Fig**). We also validated successful neural differentiation by the enrichment of neural markers including NeuN and synaptophysin using western blot (**S15F** and **S15G Fig**) [60].

Compared to transfection with a control plasmid resembling the backbone containing the Alu elements, we found that transfection of both the *circGSK3βx9-7* and *circGSK3βx10-9* isoforms resulted in reduced phospho-tau with the *circGSK3βx10-9* isoform impacting phospho-tau significantly more so than *circGSK3βx9-7* (**Figs 6E** and **S15H**). We also found transfection of both isoforms to result in reduced total GSK3β abundance and the *circGSK3βx10-9* isoform resulted in decreased phospho-Serine 9 GSK3β. When we compared the ratio of phosphorylated protein to total protein, unexpectedly we found that transfection of both *circGSK3β* isoforms resulted in reduced tau phosphorylation compared to control. However, *circGSK3βx10-9* in particular reduced tau phosphorylation significantly more than *circGSK3βx9-7* (**Fig 6F**). We did not find any significant differences in phosphorylated GSK3β. Taken together, these results demonstrate that these *circGSK3β* isoforms have contrasting impacts on tau and its phosphorylation.

## Discussion

### Synaptosome sequencing reveals RNA mislocalization as a key feature of Alzheimer’s disease

When a synapse is stimulated, rapid, local changes result in the activation of translational machinery on relevant, proximal RNAs [9–11]. Past studies have established synaptic-localized transcriptomes in cell culture and healthy models [22,61,62]. In this study, we sequenced the synaptic-localized transcriptomes of human brain tissue, and we discovered remarkable differences between AD patients and cognitively healthy, age-matched controls. We furthermore identified differences among different brain regions (frontal and temporal lobes), as well as differences from a popular mouse model of fAD. Therefore, our study reveals that the synaptic-localized transcriptome is perturbed in AD independent of global expression changes, which can impact proper localized translation and synaptic plasticity.

There may be several mechanisms responsible for this observation. One could be that there are differences in the abundance of RNA binding proteins to shuttle their respective RNA targets or that help protect mRNAs at the synapse from degradation [23,63–67]. It has been shown that dysfunction of motor proteins such as the kinesin family affects the transport of mRNAs in neurons [68]. Another could be errors in alternative splicing and post-transcriptional processing that dictate RNA destinations [18,69,70]. There is substantial evidence that alternative 3′UTRs facilitate mRNA localization [15–17,19–22]. Although our ability to detect this phenomenon was limited, we nevertheless identified enrichment of particular microRNA binding sites within the 3′UTRs of mislocalized mRNAs suggesting that the interaction between 3′UTRs and microRNAs contributes to mRNA mislocalization. Alternatively, it could be that the efficiency of RNA cytoskeletal transport itself is impaired [25,27,28]. There is evidence that each of these mechanisms are impacted in AD, though it has been unknown how they could be connected to localized translation despite observations that localized translation was impaired in AD mouse synaptosomes [71]. We show that there is at least an impact of RNA localization to synapses in AD and a collection of specific transcripts that are common among sAD patients. This finding is underscored by the fact that changes in synaptic transcripts between AD and control are not correlated to homogenate differential expression between AD and control (**Figs 2D**, **3D**, and **4D**), thus indicating that transport of RNAs play a more critical role than global differential expression in synaptic function and degeneration.

The data produced from this study adds a new dimension to our understanding of neurodegeneration and AD. Overall, synaptic localized transcripts are largely congruent between AD and control, and between different brain regions. Importantly, hierarchical clustering methods continuously differentiate synaptosome expression from homogenate expression (**S2 Fig**). The difference is the ability of these transcripts to be properly trafficked and localized to the synapse independent of global expression changes in AD. Inefficiently localized RNAs may not be translated at their expected levels thereby impacting functions taking place at the synapse [22,62,71]. For example, GO analysis among the frontal lobe mislocalized genes postulates that cell adhesion processes are largely affected (**Fig 2E** and **2F**). In addition, transcripts related to ion transport are downregulated which could provide a means of interpreting synaptic dysfunction in AD. On the other hand, GO terms among upregulated mislocalized transcripts in both the human frontal and temporal lobes relate to signaling, cell communication, and development. This finding could substantiate theories of neuronal overexcitability in AD [2,6]. Another finding is that the frontal lobe appears to be impacted differently than the temporal lobe. This is reflective of our understanding of the development and spread of AD pathology in the brain.

Something that stands out from this research is that this mouse model does not faithfully recapitulate our findings from human sAD brain tissue. There are stark contrasts between synaptic localized transcripts between wild type mice and non-demented humans, as well as contrasts in mislocalized transcripts between AD and control, perhaps reflecting species or mouse model differences. It is worth noting that the AD mouse model is more representative of fAD, as opposed to the sAD patients from which tissue was obtained considering that pathological and non-pathological features are more heterogeneous in sAD. Although mouse models have been extremely beneficial at studying hallmark aspects of AD, they may not be entirely suited for studying the complexity of mRNA transport and subsequent localized translation [72]. PMI and cause of death may also play a substantial role in transcript differences. Human samples were collected <8 hrs postmortem, while mice were euthanized via CO_2_ and dissected within 2 hrs. It is important to keep in mind that our analysis of mouse and human synaptosomes in AD may have led to the enrichment of transcriptional pathways geared towards protecting synapses in the context of extensive synaptic degeneration.

Our circRNA analysis reveals novel insights into possible mechanisms behind synaptic dysfunction. We discovered circRNAs are key regulatory molecules at synapses likely impacting protein expression. We find more than 200 circRNAs are misregulated in AD both in the frontal and temporal lobes. There are also stark differences between the brain regions. We compared our circRNA findings to the data produced from Dube et al. which was obtained from bulk homogenate [56]. We discovered considerable overlap between the data sets including *circHOMER1*, which has previously been shown to be misregulated in AD, as well as *circATRNL1*, *circMAP7*, *circRTN4*, *circMLIP*, *circFMN1*, and *circICA1* [73]. A noteworthy finding from our data is that a selection of circRNAs show contrasts in the differential expression of different isoforms arising from the same gene, including two isoforms of *circGSK3β*: an exon 9-to-7 isoform that is upregulated and a 10-to-9 isoform that is downregulated in AD. These isoforms also have contrasting regulatory effects on tau phosphorylation when overexpressed in neural cell culture. This finding, in the context of a study demonstrating oligomeric tau can be spread by way of synaptic junctions, suggests that proper balance of these circRNA isoforms may slow the spread of tau pathology between neurons [74].

### Future directions

Our discovery of two unique *circGSK3β* isoforms with differential expression in AD presents new targets for the development of AD therapeutics. We present evidence suggesting that these two isoforms play contrasting regulatory roles in the expression and phosphorylation of proteins relevant to AD progression including GSK3β and tau. Previously, attempts have been made to target GSK3β as a treatment for AD, though trials have been unsuccessful thus far [75,76]. Indeed, administration of the FDA-approved GSK3β inhibitor, valproic acid, may even worsen disease course in people with AD [77–79]. Therefore, innovative attempts to carefully restore the balance of these two isoforms may represent a potential treatment strategy. In addition, we identify hundreds of other circRNAs to be misregulated in AD, all of which could be playing consequential regulatory roles at the synapse. Our *circGSK3β* delivery experiments demonstrate successful expression and correct splicing of designed circular RNAs using a specialized plasmid created by Liang et al [59]. This paves the way for the design of viral payloads that could express circRNAs as gene therapies.

Our results also open a new avenue to test neuron and synaptic dysfunction in AD and neurodegenerative disease. Although we identify mislocalized RNAs, it remains to be seen whether these mislocalized RNAs are also improperly translated upon synaptic stimulation. A follow-up study could include a Ribo-Seq approach to identify what transcripts are being actively translated [80,81]. Ideally, metabolically active synaptosomes could be isolated and stimulated, subsequently measuring protein translation over time [71].

In conclusion, we put forth a novel RNA-seq analysis of AD from human sAD brain synaptosomes and in the APP^swe^/PSEN1^dE9^ fAD mouse model. We highlight a rigorous approach to identify mislocalized synaptic RNAs. This includes both mRNAs, as well as many circRNAs, which could both be contributing to synaptic dysfunction and loss in AD or related neurodegenerative diseases. These datasets present a launching point for additional studies into the mechanisms by which RNAs become mislocalized and their corresponding impact on localized translation and synaptic function. Finally, we provide evidence that novel therapeutic strategies could be established to express circRNAs to regulate proteins at the level of synaptic translation.

## Methods

### Ethics statement

All samples were collected following approved protocols with informed written consent as approved by the University of Washington Institutional Review Board (IRB). The University of Washington Institutional Animal Care and Use Committee (IACUC) approved all mouse studies (protocol 4387–01).

### Human brain samples

Human brain tissue samples used in this study were derived from brains donated for research by participants in the UW ADRC and the Kaiser Permanente Washington (KPW) ACT study, including 11 donors with neuropathologically-confirmed AD dementia, and 12 cognitively healthy individuals with no or low AD neuropathologic change (**S1 Table**). The ACT study is a community-based cohort study in Seattle, WA in which KPW patients 65 years or older are randomly invited to join the ACT study and, if cognitively normal, are enrolled and undergo cognitive screening every two years until they convert to dementia, withdraw from the study, or die; a subset of ACT participants consent to donate their brains for research [47]. The UW ADRC is a mostly clinic-based study of participants with varying levels of cognitive impairment who are followed annually with a battery of neuropsychometric tests until they withdraw from the study or die, and most consent to donate their brains for research. In selecting donors for this study, cognitive status and AD and related dementias neuropathologies were considered, with donors with AD dementia carefully matched with non-demented controls for age, sex, and post-mortem interval (PMI). Tissues were collected during rapid brain autopsy and flash frozen in liquid nitrogen or supercooled isopentane. For 16 of the samples, we acquired tissues from both the frontal lobe (middle frontal gyrus–dorsolateral prefrontal cortex) and temporal lobe (middle temporal gyrus) which were preserved in 0.32M sucrose, 25mM tris buffer. Other samples we collected were from the frontal lobe which were preserved by flash-freezing in isopentane.

### Mouse brain samples

Mice were maintained on an irradiated diet and provided free access to filtered water, with housing conditions maintained as previously described [82]. Following CO_2_ asphyxiation according to AVMA guidelines, brains were dissected from a mouse line bearing the APP Swedish mutation (K595N/M596L) and deletion of *PSEN1* exon 9 in the C57Bl/6 background with WT littermates used as controls [50]. Brains were sectioned based on brain regions, of which the frontal cortex was used for sequencing.

### Synaptosome isolation and RNA extraction

We adapted established protocols to isolate synaptosomes [29,30]. Approximately 300 mg of brain tissue was homogenized using a glass douncer in 1.5 mL of homogenization buffer: 0.32 M sucrose, 1 mM sodium bicarbonate, 1 mM magnesium chloride, 0.5 mM calcium chloride, 100 μM aurintricarboxylic acid, 24 U/mL NEB Murine RNAse inhibitor, and 0.5x Thermo HALT protease and phosphatase inhibitor cocktail. Following homogenization, 120 μL of homogenate was set aside for RNA extraction and sequencing and additional analyses. The remaining homogenate was spun down at 750 × g for 10 min at 4°C to remove debris. Next the supernatant was centrifuged at 13,800 × g to produce a pellet of crude synaptosomes. The supernatant was collected as the cytoplasmic fraction. The crude synaptosome pellet was resuspended in 1 mL of homogenization buffer. A discontinuous sucrose gradient was prepared in a 4.2 mL ultracentrifuge tube with a 1 mL bottom layer of 1.2 M sucrose + 1 mM sodium bicarbonate, 1 mL middle layer of 1.0 M sucrose + 1 mM sodium bicarbonate, and a 1 mL top layer of 0.85 M sucrose + 1 mM sodium bicarbonate. The 1 mL of resuspended crude synaptosome was layered on top of the gradient. The samples were then spun using a swinging bucket rotor in an ultracentrifuge at 28,000 rpm (~82,000 × g). The purified synaptosomes were collected from the interface between the 1.2 M and 1.0 M layers. RNA was subsequently extracted from 500 μl of the collected synaptosomes with Trizol/chloroform. We validated synaptosome isolation by western blot.

### Synaptosome protein analysis

Protein from the homogenate, crude P2 pellet, cytoplasm S2, and isolated synaptosome fractions were precipitated using methanol/chloroform extraction. Proteins were resolubilized in 1% SDS, 100 mM triethylammonium bicarbonate and 15 min bath sonication. Protein concentrations were estimated using Qubit (Thermo) and equal amounts were set aside for western blot loading. Blot quantification conducted with FIJI software (Image J). Antibodies: Mouse anti-actin (mAbGEa) (Invitrogen: MA51-744), Rabbit anti-NeuN (Invitrogen: PA5-78499), Rabbit anti-synaptophysin (SP11) (Invitrogen: MA5-14532), Mouse anti-IBA1 (Proteintech: 66827-1-Ig), Rabbit anti-SNAP25 (Abcam: AB108990), Rabbit anti-TDP-43 (ABClonal: A13405), Rabbit anti-RAB4 (Invitrogen: PA3-912), Rabbit anti-COX4 (Invitrogen: MA5-15078), IR dye 680 goat anti-rabbit (LI-COR: 926–68071), IR dye 800 goat anti-mouse (LI-COR: 926–32210).

### Sequencing

100 ng of RNA from synaptosome and pre-fractionated homogenate fractions were aliquoted for library preparation using the Illumina stranded total RNA with rRNA removal (human). Samples were barcoded and pooled into batches of twelve for sequencing submission to GeneWiz (Azenta) using 2 x 150 bp reads, on an Illumina NovaSeq S4 instrument. Data was delivered as fastq files pertaining to each sample. Prior to data analysis, sequencing data quality was evaluated using the fastp tool available on the Galaxy online platform. All samples passed accepted thresholds (examples in **S16A** and **S16B Fig**).

### Data analysis for localization

Transcriptome mapping and quantification was conducted with Salmon [83]. Transcriptome references were retrieved from Gencode (version 33 Homo sapiens, and version M31 Mus musculus). Salmon quant files for each sample were imported into R for differential expression analysis using the DESeq2 package and workflow [84,85]. Expression analysis was normalized based on default DESeq2 parameters which takes into account the total number of reads as well as the length of each gene. We first ran DESeq2 on a dataset of all samples with (design = ~ Fraction + Condition). We filtered our gene list by having ten counts in at least nine samples. From this, we used sample clustering and multidimensional scaling analysis to identify outliers and hierarchical clustering and weighted gene correlation analysis (WGCNA) to identify confounding variables [86]. WGCNA parameters included all defaults with adjustments as follows: maxblocksize = 10500, power = 9, corType = "bicor”, networkType = "signed", TOMType = "signed", deepSplit = 3, minModuleSize = 24, stabilityCriterion = "Individual fraction", reassignThreshold = 0.00001, and mergeCutHeight = 0.15. The single variable that stood out most was the way in which the brain tissue was preserved. Some frontal lobe samples were sucrose preserved, while some samples were preserved by isopentane; all temporal lobe samples were sucrose preserved. If we ran DESeq2 using “preservative” as our variable of comparison in synaptosome data, we saw substantial differences in RNA expression. We successfully corrected for this phenomenon using the software ComBat-Seq treating “preservative” as a batch variable [87]. This correction nearly eliminated the variation as seen before (**S17A** and **S17B Fig**). After batch correction and removing outliers, clustering of sample distances and multidimensional scaling analysis showed robust separation and clustering of synaptosomes vs homogenate and modest separation and clustering of disease status (**Figs 1D** and **S1B**). A minor confounding variable determined by WGCNA was age. We did not correct for age or incorporate it into our interpretation of results. PMI, RIN score, sex, and *APOE* genotype did not show significant effects on data clustering and not considered as confounding variables.

To conduct localization analysis on a brain region, we first created four separate datasets corresponding to control samples, AD samples, homogenate samples, and synaptosome samples. We ran DESeq2 independently on each dataset. We filtered our gene list by having ten counts in at least nine samples. Next, we applied batch correction using ComBat-Seq based on preservative for frontal lobe samples; we did not apply batch correction for temporal lobe nor mouse frontal lobe samples [87]. To identify synaptosome enriched transcripts, we ran DESeq2 differential expression analysis on either the control dataset or AD dataset using results (“Fraction”, “Synaptosome”, “Homogenate”). To identify differentially expressed genes between AD and control in either the homogenate or synaptosome datasets we ran results (“Condition”, “AD”, “Control”).

To determine mislocalized transcript differences, we first made a ratio-of-ratios comparing the synaptosome results fold change to homogenate results fold change as such:

$$Fold\;Change=\log_{2}\frac{{\left(\frac{AD}{Control}\right)}_{Synaptosome}}{{\left(\frac{AD}{Control}\right)}_{Homogenate}}$$

To construct a *p* value, we assumed a normal distribution of the synaptosome log_2_ fold change versus homogenate log_2_ fold change values from a y = x (i.e. 1:1) trendline as pictured in **Fig 2D**. We therefore calculated a difference of log_2_ fold changes of AD vs control between the synaptosome value and the homogenate value weighted by their standard error for each measured transcript. We calculated the weighted log_2_ fold change differences across all measured transcripts to produce a T-statistic based on the assumption that the weighted log_2_ fold change differences follow a normal distribution along y = x. A *p* value was drawn from a two-tailed normal distribution with N-1 degrees of freedom and setting an FDR cutoff of 0.001 for the frontal lobe and 0.01 for the temporal lobe and mice due to differences in sample sizes. Our formula for calculating a T-statistic per gene is represented by:

$$T=\frac{R_{w}}{\frac{\sqrt{\frac{\sum_{i=1}^{number\;of\;genes}{R_{w}}^{2}}{number\;of\;genes}}}{\sqrt{Number\;of\;Samples}}}$$

Where:

$$R_{w}=\frac{1}{SE}\times {\log_{2}\left(\frac{AD}{Control}\right)}_{Synaptosome}-{{\frac{1}{\mathrm{SE}}\times \log}_{2}\left(\frac{AD}{Control}\right)}_{Homogenate}$$

R_w_ represents the difference in log_2_ fold changes of AD vs control weighted by their respective standard errors between. Since DESeq2 calculates differential expression using a Wald statistic, our approach could be interpreted as simply taking a T-statistic between the Wald statistics. This resulted in a selection of significantly measured mislocalized mRNAs representing the expected fraction of a transcript’s reads found in synaptosomes compared to homogenate is different in AD versus control. This could represent errors specifically in RNA transport or degradation dynamics within the neurites independent of global expression changes.

Gene ontology analysis was conducted using GOpanther “biological process complete” [88]. Genome alignments for visualization were conducted using RNA-STAR [89]. Human genomes were aligned to GRCh38 and mouse genomes were aligned to GRCm39 acquired from Ensembl.

### CircRNA identification

We generated a custom back-spliced junction database by adding all possible permutations of exon combinations in which a downstream exon’s 3′ end, the “Donor”, is joined to the 5′ end of an upstream exon, the “Acceptor”. Next, we generated bowtie2 indexes for both the back splice junction file and the Gencode v. 33 reference transcriptomes. The samples’ fastq files were first mapped to the reference canonical transcriptome using bowtie2 [90]. Unmapped reads were extracted from the resultant alignment files using samtools [91]. The unmapped read files were then converted into fastq files using bedtools [92]. Next these fastq files were mapped to the back splice junction index with bowtie2. CircRNA read alignments were counted for each sample producing a count matrix. This count matrix was then appended to the count matrix of linear canonical transcript unnormalized counts and imported back into R to run DESeq2 differential expression analysis on the combined counts to normalize in context of the rest of the transcriptome. We filtered our circRNA list by having four counts per unique circRNA in at least five samples. To test our approach against DCC, we used the standard workflow and settings including the preliminary alignment using RNA-STAR [55].

### miRNA site analysis

To identify miRNA binding sites, the biomaRt package was used to obtain 3′UTR sequences for all genes we measured which were then input into Targetscan producing a result table counting all miRNA binding sites within each UTR across the transcriptome [93,94]. To predict miRNA binding site enrichment among significantly mislocalized mRNAs, the UTRs were retrieved for our short-list and using Targetscan v. 7.0, and we produced a count table of all miRNA binding sites. Using our transcriptome-wide miRNA site index as a background, we calculated the expected values for each miRNA binding site distributed among our short-lists. We calculated a log_2_ fold change based on the ratio of observed vs expected fraction each miRNA was represented out of the total number of identified binding sites (i.e. the fraction of total binding sites for a miRNA found within our short list versus the fraction of total binding sites for said miRNA throughout the entire transcriptome). We calculated significance of miRNA binding site enrichment using a χ^2^ test.

### PCR validation

PCR amplicons for circRNAs were designed with primers that would amplify the back splice junction. We then Sanger sequenced these amplicons to validate the junctions. Primers are listed in **S32 Table**.

### Circ plasmids

We ordered custom synthesized plasmids from Genscript designed with *GSK3β* exon inserts between Alu elements in the pcDNA3.1+ backbone designed, proven, and published to coax RNA circularization (Addgene #60648) [59].

### Transfections

SH-Sy5y cells, obtained from ATCC, were differentiated into neurons using an abbreviated protocol based on Shipley et al [60]. Undifferentiated cells were cultured in a 50/50 mixture of DMEM/F12 media supplemented with 15% heat inactivated fetal bovine serum (FBS) plus 1% penicillin/streptomycin solution. Undifferentiated cells were passaged into T25 flasks to begin differentiation on day 0. On day 1, media was swapped to 3% FBS solution supplemented with 10 μM all-trans retinoic acid. On day 3, media was swapped to 1% FBS solution supplemented with 10 μM all-trans retinoic acid. On day 5 cells were passaged onto Geltrex (Gibco) coated 6 well plates, 120,000 cells each. On day 6, media was refreshed, and transfections took place using lipofectamine using 1.5 μg plasmid with 5 μl per well in 250 μl Opti-MEM. On day 7, media was swapped to Neurobasal plus media supplemented with 1x B27 plus, 2 mM Glutamax, 1% penicillin/streptomycin, 20 mM potassium chloride, 50 ng/mL BDNF, 2 mM dibutyryl cyclic AMP, and 10 μM all-trans retinoic acid. Media was refreshed on day 9, day 11, and day 12. Cells were harvested on day 13 with 1 mL RIPA buffer and sonicated in a bath at 4°C for 15 min. Protein was quantified with qubit for equal loading in western blot. Blot quantification conducted with FIJI software (Image J). We validated circular RNA formation using PCR and Sanger sequencing where we confirmed bands associated with the complete circle and proper splicing. Antibodies include: rabbit anti-GSK3β (Cell systems: D5C5Z), rabbit anti-pS9-GSK3β (Cell systems: D85E12), mouse anti-pS202,T205-tau (AT8) (Invitrogen: MN1020), mouse anti-tau (Tau5) (Invitrogen: AHB0042), rabbit anti-GAPDH (Cell Systems: 14C10), Mouse anti-beta tubulin (BT7R) (Invitrogen: MA5-16308), Rabbit anti-NeuN (Invitrogen: PA5-78499), Rabbit anti-synaptophysin (SP11) (Invitrogen: MA5-14532), IR dye 680 goat anti-mouse (LI-COR: 926–68070), IR dye 680 goat anti-rabbit (LI-COR: 926–68071), IR dye 800 goat anti-mouse (LI-COR: 926–32210), IR dye 800 goat anti-rabbit (LI-COR: 926–32211).

### BaseScope in situ hybridization

Custom BaseScope probes spanning the *GSK3β* circRNA exon 10-to-9 and exon 9-to-7 junctions of NM_002093.4 were designed and ordered by ACD. In addition, we designed a similar probe to the mRNA sequence of *GSK3β* that is not part of the circRNA. The BaseScope duplex reagent kit and protocol was used for hybridization. Brain slices of a square area approximately 0.3 cm x 0.3 cm from our frontal lobe sample collection were sectioned via cryostat into 20 μm sections onto Superfrost Plus slides. Brain tissue slides were fixed using chilled 4% formaldehyde in PBS for 15 min. Next, the slides were dehydrated by a 5 min wash with 50% ethanol, a 5 min wash with 70% ethanol, and two 5 min washes in 100% ethanol. Slides were dried at room temp before proceeding with the BaseScope Duplex protocol. The only modification to the protocol was extending the AMP step [11] to 40 min instead of 30 min. *CircGSK3β* exon 9-to-7 and *CircGSK3β* 10-to-9 were visualized using Fast-Green chromogenic reagent. Linear *GSK3β* visualized using Fast-Red chromogenic reagent. Nuclear and membrane stained using 0.5x Gil’s hematoxylin. Slides were imaged in brightfield on a Nikon Ti-2 microscope. Quantification was done by research scientists blinded to the hypothesized outcome who counted RNAs from 8 or more images per slice for several tissue slices.

### Synaptosome confocal microscopy

We followed a modified protocol previously utilized for synaptosome immunohistochemistry [48]. First, bovine serum albumin coated slides were prepared by immersing the slides in 3% BSA, PBS and sodium azide overnight at 4°C. Slides were subsequently washed with PBS and left to dry. 0.5 cm x 0.5 cm squares were drawn on the slides with nail polish. 250 μL synaptosomes were diluted 5x in PBS in microcentrifuge tubes. They were spun down at 12,000 × g for 20 min at 4°C. The synaptosome pellet was resuspended in 50ul PBS. 5ul of resuspended synaptosomes were added to each outlined square on the BSA coated slides. This was followed by the addition of 5 μl of PBS. Next, 10 μl of 8% formaldehyde was added to the synaptosomes on the square. Synaptosomes were allowed to fix at room temperature for 1 hour. Next, excess liquid was aspirated from the squares leaving synaptosomes fixed on the slides. Excess formaldehyde was quenched with 35 μl of 100 mM glycine. Excess liquid was aspirated. Synaptosomes were stained with the lipophilic fluorophore FM1-43, 20 μl at 5 μg/mL concentration for 5 min at room temperature. Excess stain was aspirated. Stained synaptosomes were washed with 35 μl PBS for 10 min at room temperature. Excess liquid was aspirated. Drops of Vectashield mounting media were placed within the squares and coverslips placed on top. Coverslips were sealed with nail polish. Slides were imaged on an Olympus FV-1000 confocal in the GFP channel.

## Supporting information

S1 Fig Extended data for Fig 1; Human frontal lobe sample properties. **A.** Additional western blots probing subsequent fractionation steps of control samples during synaptosome preparation for TDP-43, a synaptic marker, SNAP25, an endosomal marker, RAB4, and a mitochondrial marker, COX4, with Actin as a loading control. **B.** Quantification of western blots in (**A**). **C.** Western blot of a synaptic marker, synaptophysin, a nuclear marker, NeuN, and a microglial marker, IBA1, with Actin as a loading control for the different fractionation steps during synaptosome preparation in AD samples (See **Fig 1B** for control samples). **D.** Fluorescent membrane staining and confocal imaging of the synaptosomal fraction shows intact bipartite structures of expected size. **E.** Sample distance heatmap showing robust clustering of synaptosome and homogenate fractions among AD and control frontal lobe samples. **F.** Heatmap substantiating robust clustering of synaptosome and homogenate fractions as well as minimal impact of other variables after batch correction including PMI, RIN, sex, and *APOE* alleles. **G.** Volcano plot comparing synaptosome vs homogenate in AD samples shows the same pattern as control samples in (See **Fig 1E**).
(TIF)

S2 Fig Extended data for Fig 1; Additional multidimensional scaling plots. **A.** Multidimensional scaling analysis among control samples only substantiating that most sample variation is determined by synaptosome vs. homogenate fraction. **B**. The same phenomenon is apparent among only AD samples. **C-D**. Multidimensional scaling analysis among only homogenate, and only synaptosome, respectively, reveals greater separation between control and AD among the synaptosome fraction compared to homogenate.
(TIF)

S3 Fig Extended data for Fig 1; Fus alternative splicing in synaptosomes. Integrated genome viewer (IGV) version 2.16.2 image of control and AD homogenate and synaptosome RNA-seq tracks spanning the Fused in Sarcoma (FUS) locus (GRCh38 chr16:31,179,688–31,195,372) and demonstrating intron 6 and intron 7 retention events specific to homogenate samples.
(TIF)

S4 Fig Extended data for Fig 2; Expression patterns of mislocalized mRNAs in the human frontal lobe. **A.** Heatmap of significantly mislocalized mRNAs in the frontal lobe (see **Fig 2D**). Expression patterns reveal that mislocalized mRNAs in the synaptosome fraction cluster disease condition more robustly than expression patterns in the homogenate fraction demonstrating independence from global expression changes. Furthermore, other variables seem to have minimal bearing on clustering.
(TIF)

S5 Fig Extended data for Fig 3; Human temporal lobe sample properties. **A.** Sample distance heatmap showing robust clustering of synaptosome and homogenate fractions among AD and control temporal lobe samples. **B.** Multidimensional scaling analysis shows robust separation between mRNA counts of homogenate and synaptosome fractions among temporal lobe samples as well as moderate separation between disease condition. **C.** Sample distance heatmap including both frontal and temporal lobe samples showing more similarity between fractions than either tissue or disease condition.
(TIF)

S6 Fig Extended data for Fig 3; Heatmap of human temporal lobe gene expression. **A.** Heatmap including both frontal and temporal lobe samples (sucrose preservative only) showing that fraction and disease condition precedes brain region in clustering impact with minimal effects from other variables.
(TIF)

S7 Fig Extended data for Fig 3; Expression patterns in the human temporal lobe. **A.** Volcano plot of synaptosome-enriched mRNAs in control temporal lobe samples. **B.** Comparison of synaptosome-enrichment between AD and control temporal lobe samples shows strong correlation (R^2^ = 0.8955). **C.** Venn diagram showing substantial overlap of synaptosome-enriched mRNAs between AD and control temporal lobe samples (*p* < 0.01). **D.** Volcano plot comparing expression differences between AD and control in the bulk, unfractionated homogenate. **E.** Volcano plot comparing expression differences between AD and control just within the synaptosome particles. **F.** Venn diagram showing minimal overlap of differentially expressed mRNAs in temporal lobe synaptosome and homogenate fractions (*p* < 0.05). Those that do overlap have 99% concordant log_2_ fold change. **G.** Heatmap of significantly mislocalized mRNAs in the temporal lobe (See **Fig 3D**). Expression patterns reveal that disease condition has a stronger bearing on mislocalized mRNAs in the synaptosome fraction compared to the homogenate fraction demonstrating independence from global expression changes. Additionally, other variables seem to have minimal bearing on clustering.
(TIF)

S8 Fig Extended data for Fig 4; Mouse frontal lobe sample properties. **A.** Table showing sample properties of mice used for synaptosome sequencing. **B.** Sample distance heatmap shows robust separation between synaptosome and homogenate fractions. **C.** Multidimensional scaling analysis also shows robust separation between synaptosome and homogenate fractions, yet minimal separation between AD and wild-type samples.
(TIF)

S9 Fig Extended data for Fig 4; Expression patterns in the mouse frontal lobe. **A.** Volcano plot showing synaptosome-enriched mRNAs in control mouse frontal lobe samples. **B.** Comparison of synaptosome-enrichment (*p* < 0.01) between AD and control mouse frontal lobe samples shows strong correlation (R^2^ = 0.8913). **C.** Volcano plot comparing expression differences between AD and control in the bulk, unfractionated homogenate. **D.** Volcano plot comparing expression differences between AD and control just within the synaptosome particles. **E.** Venn diagram showing minimal overlap of differentially expressed mRNAs in mouse frontal lobe synaptosome and homogenate fractions (*p* < 0.05). Those that do overlap have 92% concordant log_2_ fold change.
(TIF)

S10 Fig Comparison of mouse synaptosome data with a previous study from Niu et al [37]. **A.** Comparing the fold changes/enrichment of synaptosome vs homogenate between our data and Niu et al. shows a moderate correlation (R^2^ = 0.5816). **B.** Venn diagram shows plenty of synaptosome enriched genes overlap between our data and Niu et al. **C.** Comparing the fold changes of AD vs control between synaptosomes and homogenate in Niu et al.’s data shows moderate correlation (R^2^ = 0.5062). Genes that have a T-statistic most distant from a y = x trendline are significantly mislocalized, independent of global expression changes (*p* < 0.05).
(TIF)

S11 Fig Mislocalized mRNAs show specific enrichment and de-enrichment of microRNA binding sites. **A.** Volcano plot showing enrichment of microRNA binding sites among the upregulated mislocalized mRNAs in the human frontal lobe (*p* < 0.05, see **Fig 2D**). **B.** Volcano plot showing enrichment of microRNA binding sites among the downregulated mislocalized mRNAs in the human frontal lobe (*p* < 0.05, see **Fig 2D**). **C.** Volcano plot showing enrichment of microRNA binding sites among the upregulated mislocalized mRNAs in the human temporal lobe (*p* < 0.05, see **Fig 3D**). **D.** Volcano plot showing enrichment of microRNA binding sites among the downregulated mislocalized mRNAs in the human temporal lobe (*p* < 0.05, see **Fig 3D**).
(TIF)

S12 Fig Extended data for Fig 5; CircRNA expression and properties in the human frontal lobe. **A.** Volcano plot comparing circRNAs in synaptosome vs homogenate in AD frontal lobe samples shows that the majority of circRNAs are significantly enriched at synaptic terminals. **B.** Comparing circRNAs in synaptosome vs homogenate in both AD and control frontal lobe samples shows that synaptic enrichment of circRNAs is a shared phenomenon. **C.** Multidimensional scaling plot of samples used for circRNA analysis representing the variation exclusively among circRNA reads (this is separate from the differential expression analysis where the combination of linear and circular reads were used for normalization). **D.** Analyzing the difference between the donor and acceptor exons involved in back-splicing shows that the majority of circRNAs consist of 2–3 exons.
(TIF)

S13 Fig Extended data for Fig 5; CircRNA expression in the human temporal lobe. **A.** Volcano plot comparing circRNAs in synaptosome vs homogenate in control temporal lobe samples shows that the majority of circRNAs are significantly enriched at synaptic terminals. **B.** Comparing circRNAs in synaptosome vs homogenate in both control frontal lobe and temporal lobe samples shows that synaptic enrichment of circRNAs is similar in both tissues. **C.** Multidimensional scaling plot of samples used for circRNA analysis representing the variation exclusively among circRNA reads (this is separate from the differential expression analysis where the combination of linear and circular reads were used for normalization). **D**. Volcano plot comparing circRNAs in AD vs control in the unfractionated homogenate (*p* < 0.05). **E.** Volcano plot comparing circRNAs in AD vs control in the synaptosome fraction (*p* < 0.05). **F.** Venn diagram shows minimal overlap of differentially expressed circRNAs between synaptosome and homogenate fractions with more occurring in the synaptosome fraction (*p* < 0.05). Those that do overlap show 100% concordant Log_2_ fold change. **G.** Venn diagram shows minimal overlap of differentially expressed circRNAs in the synaptosome fraction between human frontal and temporal lobes (*p* < 0.05).
(TIF)

S14 Fig Extended data for Fig 5; CircRNA expression in the mouse frontal lobe. **A.** Volcano plot comparing circRNAs in synaptosome vs homogenate in control mouse samples continues to show synaptic enrichment of circRNAs. **B.** Volcano plot comparing circRNAs in AD vs control in mouse synaptosomes (*p* <0.05).
(TIF)

S15 Fig Extended data for Fig 6; SH-Sy5y differentiation into neurons and *CircGSK3β* transfections. **A.** Sanger Sequencing confirmation of back-splice junction between exons 5 and 2 of *HOMER1*. **B.** BaseScope RNA hybridization probes show that *circGSK3β* isoforms (green) are more frequently distal to the nucleus than the linear *GSK3β* transcript (red) in human control frontal lobe brain slices. Tissue and nuclei were stained with hematoxylin. **C.** Pie charts representing quantification of nuclear versus non-nuclear localization of linear *GSK3β* and circular isoforms in BaseScope images (not significant). D. Quantification of *GSK3β* circular isoforms in AD vs control in BaseScope images (not significant). **E.** Bright field microscopy of neuron-differentiated SH-Sy5y cells confirms development of neuron morphology including polarized cells, axons, and dendrites. **F.** SH-Sy5y neuron differentiation confirmed by western blot showing an enrichment of two neuronal markers, Synaptophysin and NeuN. **G.** Quantification of blots in (**S15F Fig**). N = 6 samples per group. **: *p* < 0.01, ***: *p* < 0.001. **H.** Quantification of blots in (**Fig 6E**). N = 8 samples per group Significance calculated by T-test. *: *p* < 0.05, **: *p* < 0.01, ***: *p* < 0.001, ****: *p* < 0.0001.
(TIF)

S16 Fig Quality assessment of sequencing. Screenshots from the output of quality assessment tool, Fastp, found on the Galaxy online resource. **A.** Representative quality assessment of a homogenate sample. **B**. Representative quality assessment of a synaptosome sample [95].
(TIF)

S17 Fig Brain tissue preservation method has a major impact on mRNA measurements and can be successfully corrected for. **A.** Volcano plots comparing isopentane vs sucrose tissue preservation among both AD and control frontal lobe synaptosome samples before and after batch correction. ComBat_seq appears to substantially reduce the variation among samples based on this variable [87]. **B.** Weighted gene correlation network analysis (WGCNA) among human AD and control frontal lobe homogenate and synaptosome samples identifies confounding variables before and after ComBat_seq batch correction which shows that batch correction almost completely removes gene expression modules that correlate with preservation method with little impact on the expression variation defined by modules correlated with other variables including fraction and condition.
(TIF)

S1 Table Human and Mouse Samples. (XLSX)

S2 Table Synaptic-Enriched mRNAs, Human Frontal Lobe, Control. (XLSX)

S3 Table Synaptic-Enriched mRNAs, Human Frontal Lobe, AD. (XLSX)

S4 Table AD vs Control, Human Frontal Lobe, Homogenate. (XLSX)

S5 Table AD vs Control, Human Frontal Lobe, Synaptosome. (XLSX)

S6 Table Mislocalized Human Frontal Lobe mRNAs, AD vs Control. (XLSX)

S7 Table Synaptic-Enriched mRNAs, Human Temporal Lobe, Control. (XLSX)

S8 Table Synaptic-Enriched mRNAs, Human Temporal Lobe, AD. (XLSX)

S9 Table AD vs Control, Human Temporal Lobe, Homogenate. (XLSX)

S10 Table AD vs Control, Human Temporal Lobe, Synaptosome. (XLSX)

S11 Table Mislocalized Human Temporal Lobe mRNAs, AD vs Control. (XLSX)

S12 Table Synaptic-Enriched mRNAs, Mouse Frontal Lobe, Control. (XLSX)

S13 Table Synaptic-Enriched mRNAs, Mouse Frontal Lobe, AD. (XLSX)

S14 Table AD vs Control, Mouse Frontal Lobe, Homogenate. (XLSX)

S15 Table AD vs Control, Mouse Frontal Lobe, Synaptosome. (XLSX)

S16 Table Mislocalized Mouse Frontal Lobe mRNAs, AD vs Control. (XLSX)

S17 Table MiR Binding Sites, Frontal Lobe Upregulated. (XLSX)

S18 Table MiR Binding Sites, Frontal Lobe Downregulated. (XLSX)

S19 Table MiR Binding Sites, Temporal Lobe Upregulated. (XLSX)

S20 Table MiR Binding Sites, Temporal Lobe Downregulated. (XLSX)

S21 Table Synaptosome vs Homogenate CircRNAs, Human Frontal Lobe, Control. (XLSX)

S22 Table Synaptosome vs Homogenate CircRNAs, Human Frontal Lobe, AD. (XLSX)

S23 Table AD vs Control CircRNAs, Human Frontal Lobe, Homogenate. (XLSX)

S24 Table AD vs Control CircRNAs, Human Frontal Lobe, Synaptosome. (XLSX)

S25 Table Synaptosome vs Homogenate CircRNAs, Human Temporal Lobe, Control. (XLSX)

S26 Table AD vs Control CircRNAs, Human Temporal Lobe, Homogenate. (XLSX)

S27 Table AD vs Control CircRNAs, Human Temporal Lobe, Synaptosome. (XLSX)

S28 Table Synaptosome vs Homogenate CircRNAs, Mouse Frontal Lobe, Control. (XLSX)

S29 Table AD vs Control CircRNAs, Mouse Frontal Lobe, Synaptosome. (XLSX)

S30 Table DCC results: Synaptosome vs Homogenate CircRNAs, Human Frontal Lobe, Control. (XLSX)

S31 Table DCC results: AD vs Control CircRNAs, Human Frontal Lobe, Synaptosome. (XLSX)

S32 Table Primers for circGSK3β amplification. (XLSX)
