# Supplementary figures and images for: mRNA and circRNA mislocalization to synapses are key features of Alzheimer’s disease
Source: PLoS Genet. 2024 Jul 29;20(7):e1011359. doi: 10.1371/journal.pgen.1011359 (PMC11309398; doi:10.1371/journal.pgen.1011359)

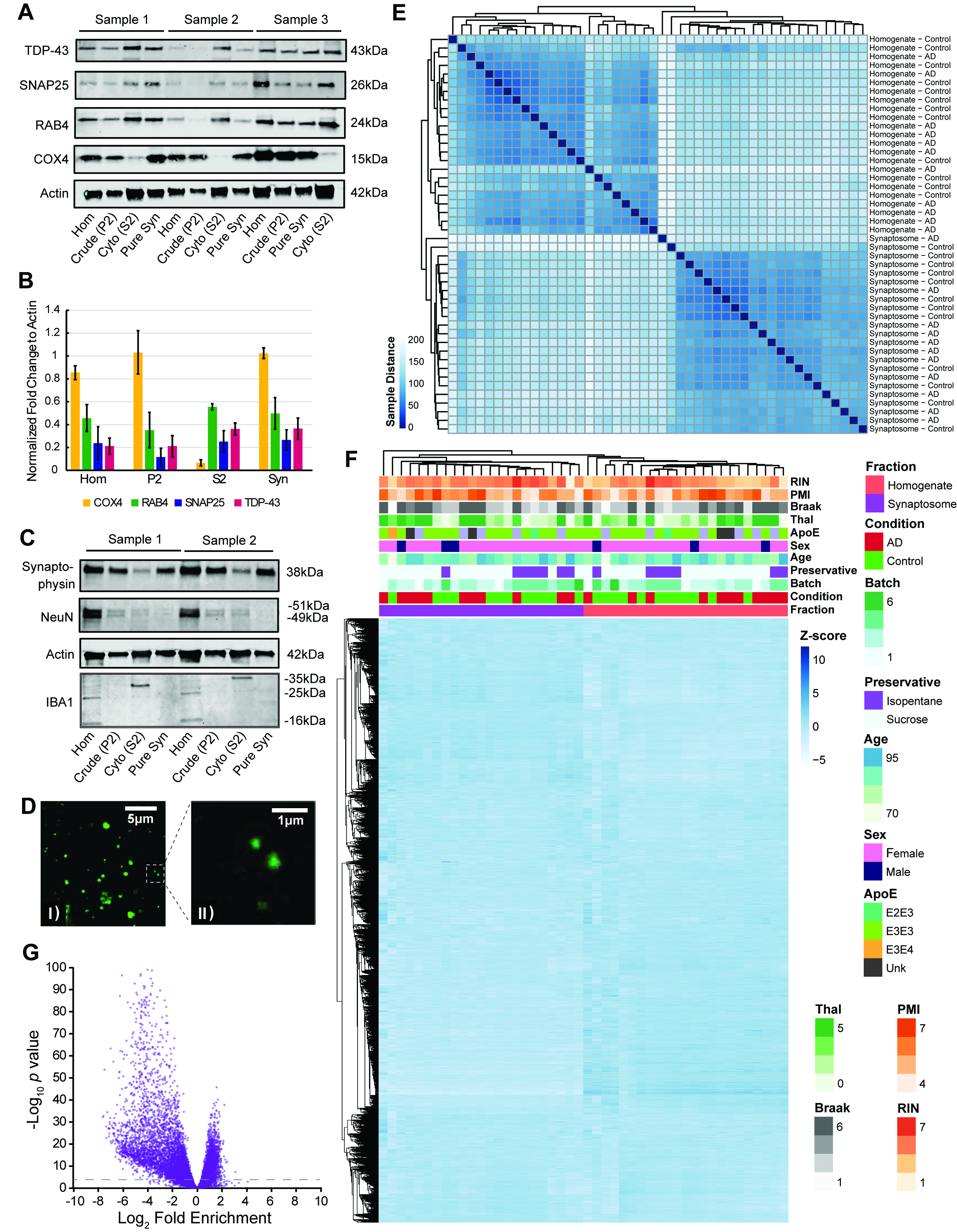

Supplement: S1 Fig — A. Additional western blots probing subsequent fractionation steps of control samples during synaptosome preparation for TDP-43, a synaptic marker, SNAP25, an endosomal marker, RAB4, and a mitochondrial marker, COX4, with Actin as a loading control. B. Quantification of western blots in (A). C. Western blot of a synaptic marker, synaptophysin, a nuclear marker, NeuN, and a microglial marker, IBA1, with Actin as a loading control for the different fractionation steps during synaptosome preparation in AD samples (See Fig 1B for control samples). D. Fluorescent membrane staining and confocal imaging of the synaptosomal fraction shows intact bipartite structures of expected size. E. Sample distance heatmap showing robust clustering of synaptosome and homogenate fractions among AD and control frontal lobe samples. F. Heatmap substantiating robust clustering of synaptosome and homogenate fractions as well as minimal impact of other variables after batch correction including PMI, RIN, sex, and APOE alleles. G. Volcano plot comparing synaptosome vs homogenate in AD samples shows the same pattern as control samples in (See Fig 1E). (TIF) [file pgen.1011359.s001.tif]

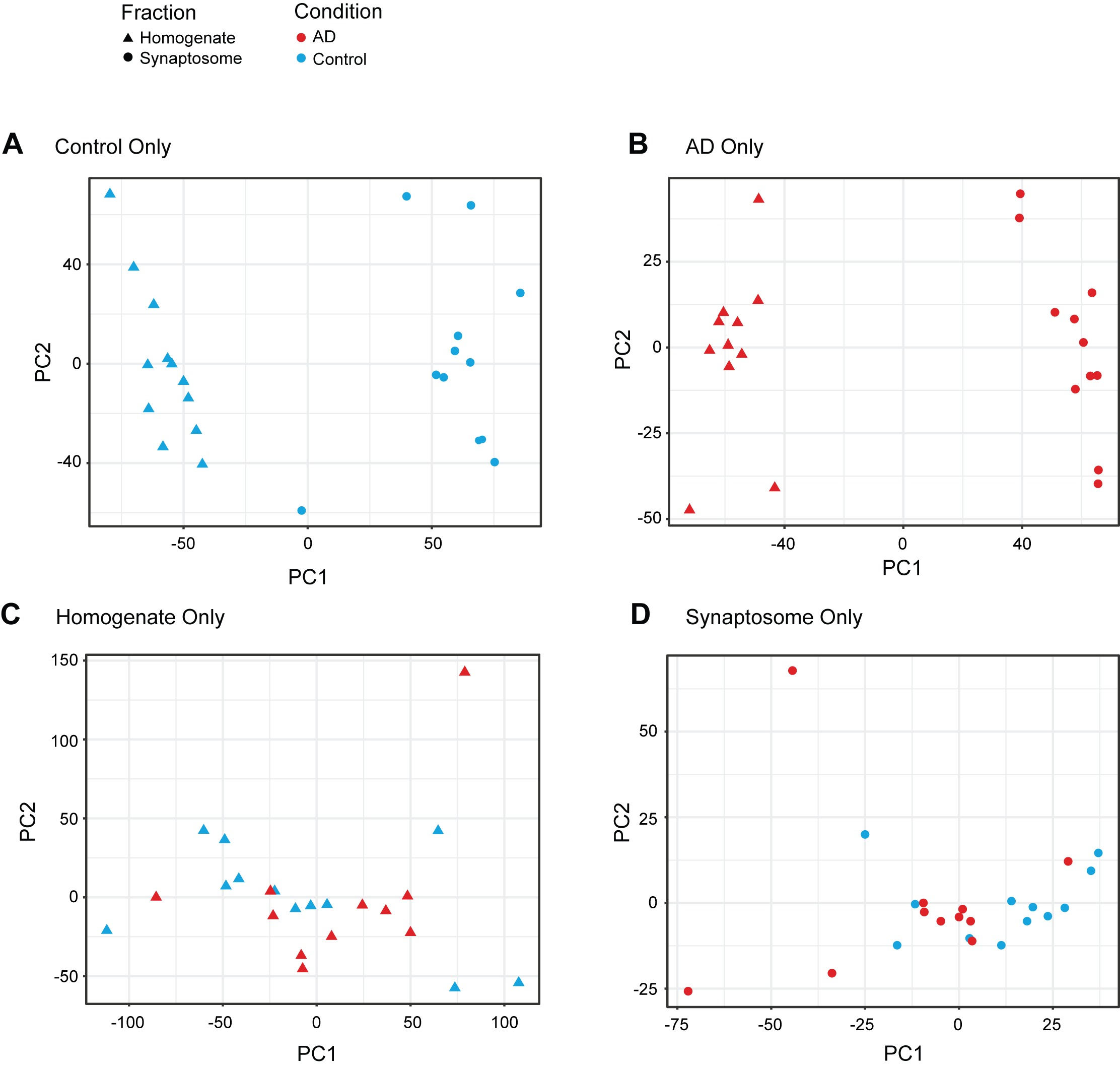

Supplement: S2 Fig — A. Multidimensional scaling analysis among control samples only substantiating that most sample variation is determined by synaptosome vs. homogenate fraction. B. The same phenomenon is apparent among only AD samples. C-D. Multidimensional scaling analysis among only homogenate, and only synaptosome, respectively, reveals greater separation between control and AD among the synaptosome fraction compared to homogenate. (TIF) [file pgen.1011359.s002.tif]

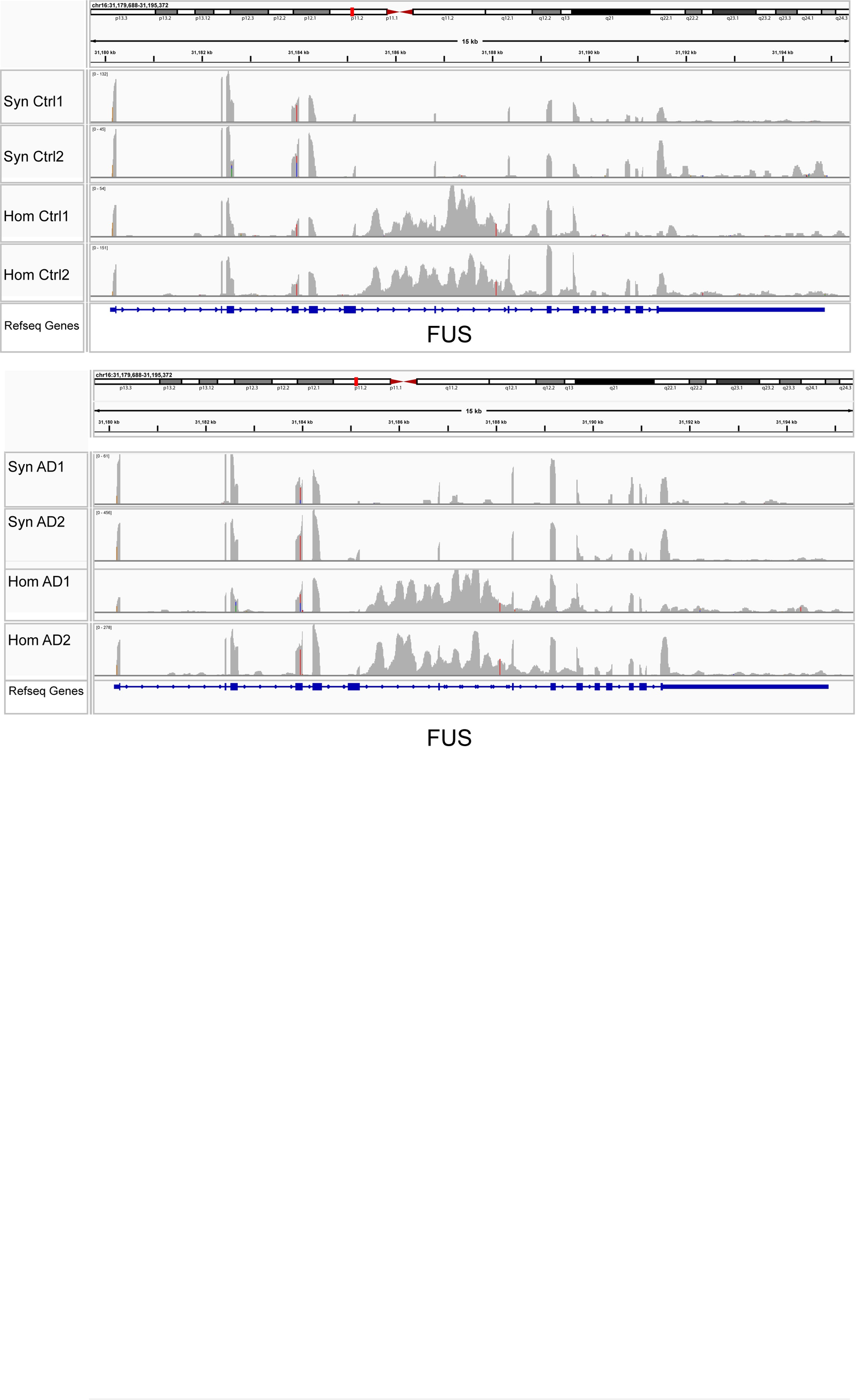

Supplement: S3 Fig — Integrated genome viewer (IGV) version 2.16.2 image of control and AD homogenate and synaptosome RNA-seq tracks spanning the Fused in Sarcoma (FUS) locus (GRCh38 chr16:31,179,688–31,195,372) and demonstrating intron 6 and intron 7 retention events specific to homogenate samples. (TIF) [file pgen.1011359.s003.tif]

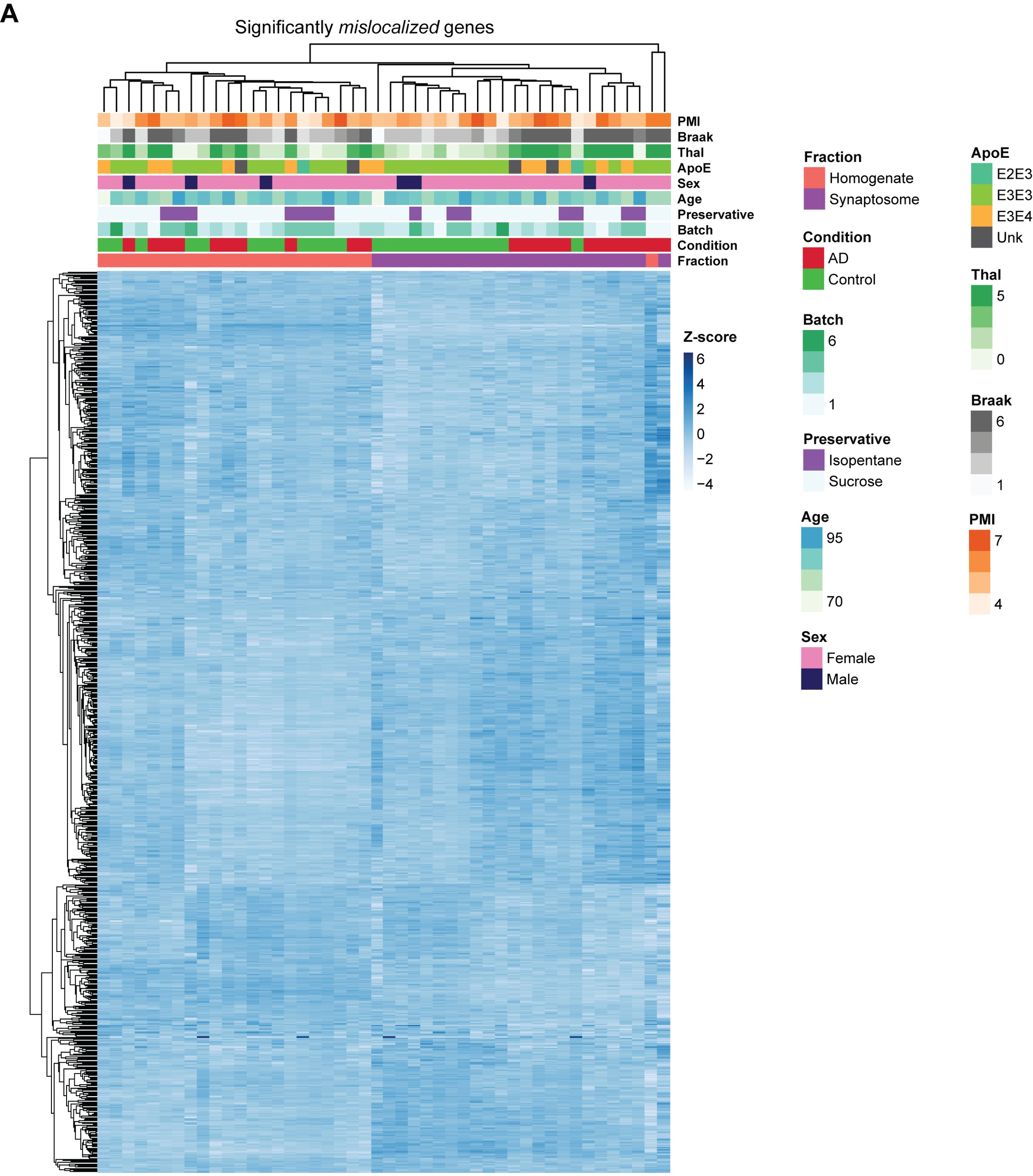

Supplement: S4 Fig — A. Heatmap of significantly mislocalized mRNAs in the frontal lobe (see Fig 2D). Expression patterns reveal that mislocalized mRNAs in the synaptosome fraction cluster disease condition more robustly than expression patterns in the homogenate fraction demonstrating independence from global expression changes. Furthermore, other variables seem to have minimal bearing on clustering. (TIF) [file pgen.1011359.s004.tif]

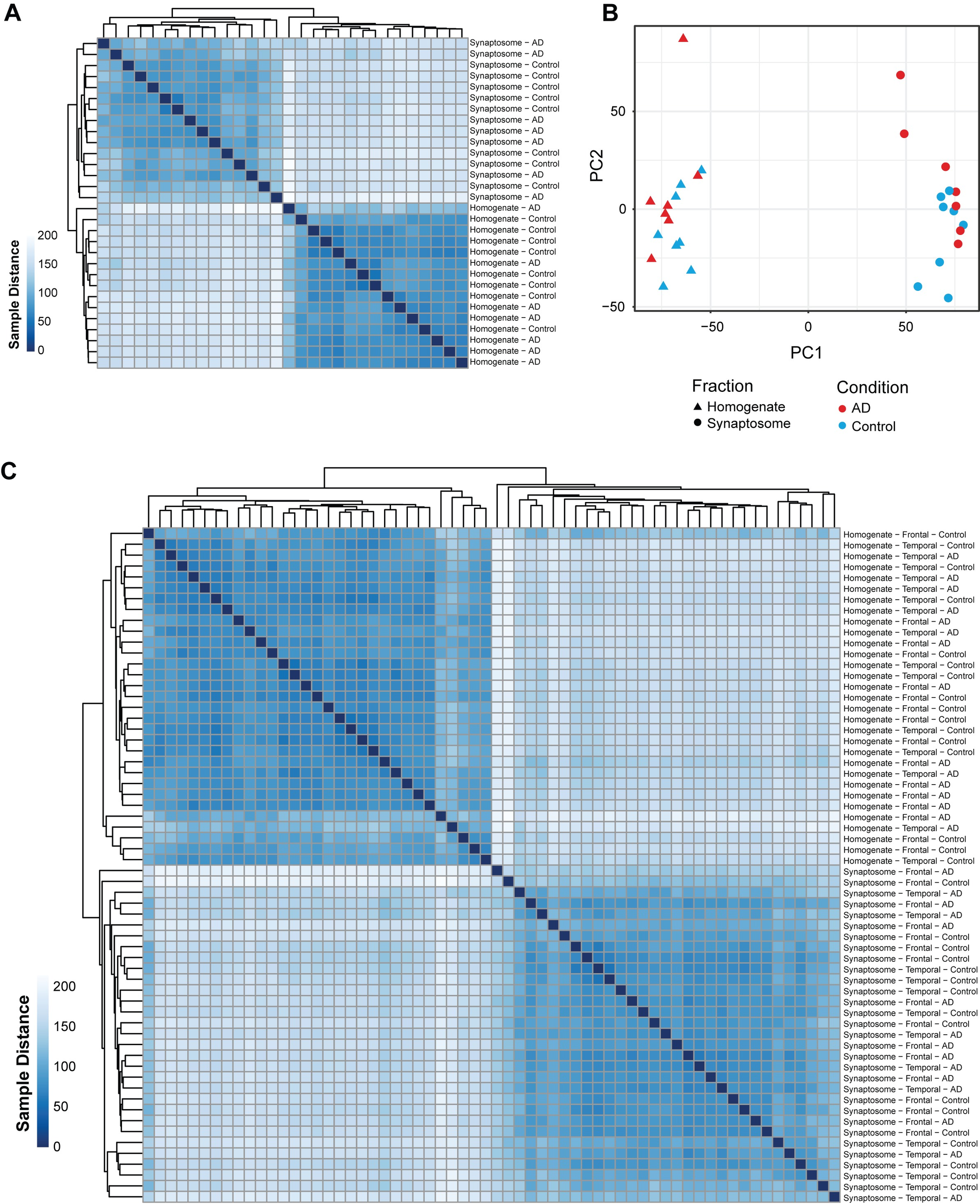

Supplement: S5 Fig — A. Sample distance heatmap showing robust clustering of synaptosome and homogenate fractions among AD and control temporal lobe samples. B. Multidimensional scaling analysis shows robust separation between mRNA counts of homogenate and synaptosome fractions among temporal lobe samples as well as moderate separation between disease condition. C. Sample distance heatmap including both frontal and temporal lobe samples showing more similarity between fractions than either tissue or disease condition. (TIF) [file pgen.1011359.s005.tif]

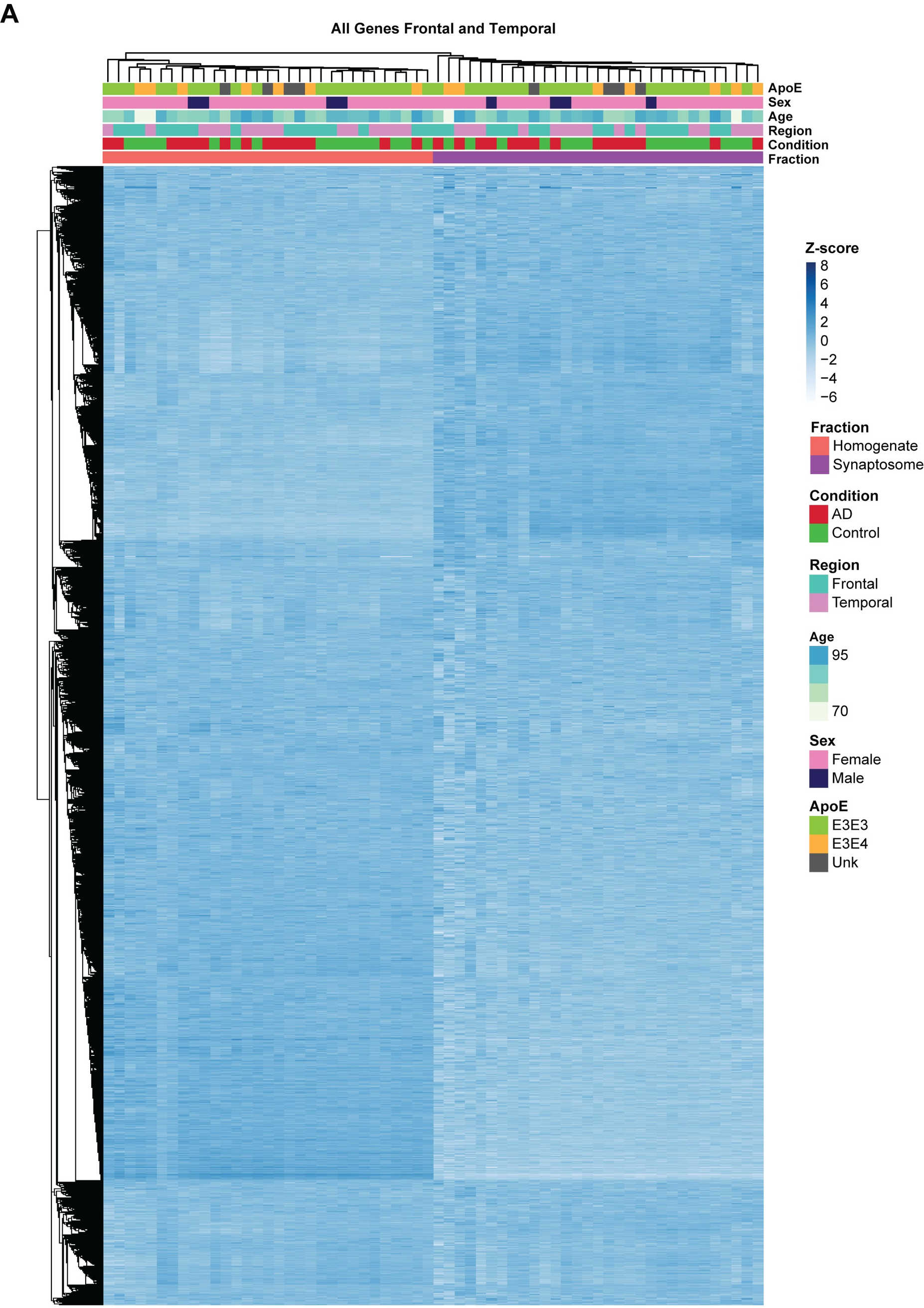

Supplement: S6 Fig — A. Heatmap including both frontal and temporal lobe samples (sucrose preservative only) showing that fraction and disease condition precedes brain region in clustering impact with minimal effects from other variables. (TIF) [file pgen.1011359.s006.tif]

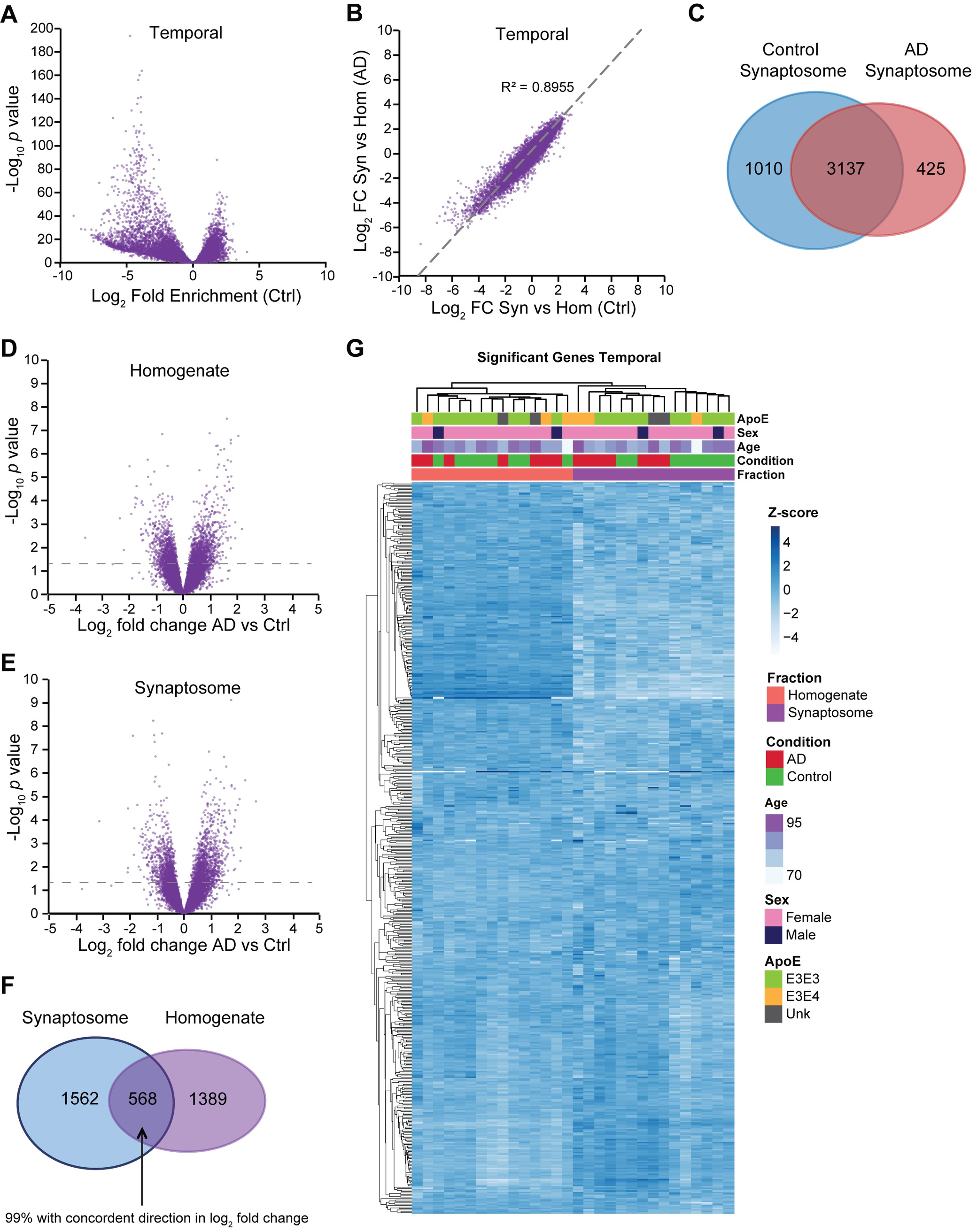

Supplement: S7 Fig — A. Volcano plot of synaptosome-enriched mRNAs in control temporal lobe samples. B. Comparison of synaptosome-enrichment between AD and control temporal lobe samples shows strong correlation (R2 = 0.8955). C. Venn diagram showing substantial overlap of synaptosome-enriched mRNAs between AD and control temporal lobe samples (p < 0.01). D. Volcano plot comparing expression differences between AD and control in the bulk, unfractionated homogenate. E. Volcano plot comparing expression differences between AD and control just within the synaptosome particles. F. Venn diagram showing minimal overlap of differentially expressed mRNAs in temporal lobe synaptosome and homogenate fractions (p < 0.05). Those that do overlap have 99% concordant log2 fold change. G. Heatmap of significantly mislocalized mRNAs in the temporal lobe (See Fig 3D). Expression patterns reveal that disease condition has a stronger bearing on mislocalized mRNAs in the synaptosome fraction compared to the homogenate fraction demonstrating independence from global expression changes. Additionally, other variables seem to have minimal bearing on clustering. (TIF) [file pgen.1011359.s007.tif]

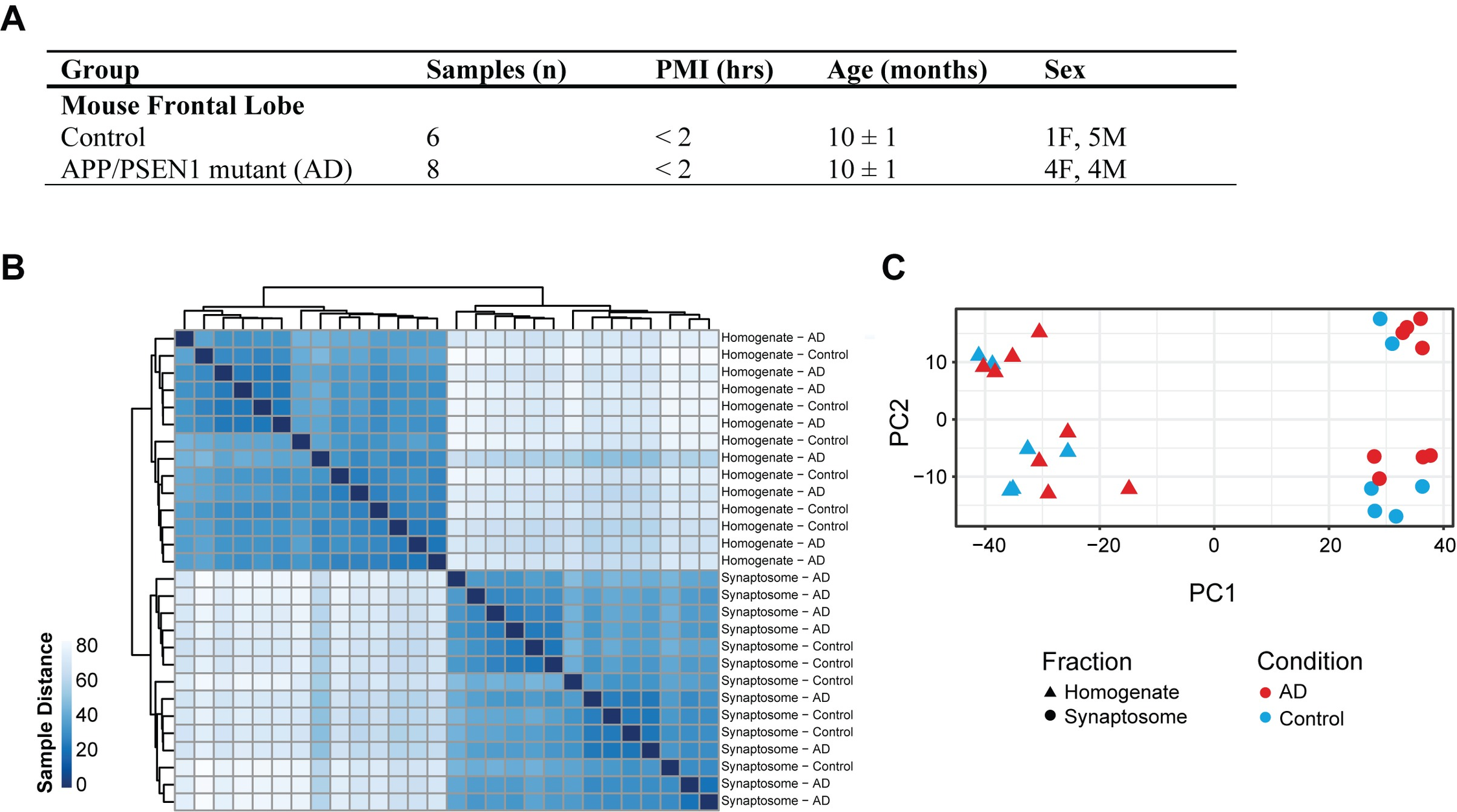

Supplement: S8 Fig — A. Table showing sample properties of mice used for synaptosome sequencing. B. Sample distance heatmap shows robust separation between synaptosome and homogenate fractions. C. Multidimensional scaling analysis also shows robust separation between synaptosome and homogenate fractions, yet minimal separation between AD and wild-type samples. (TIF) [file pgen.1011359.s008.tif]

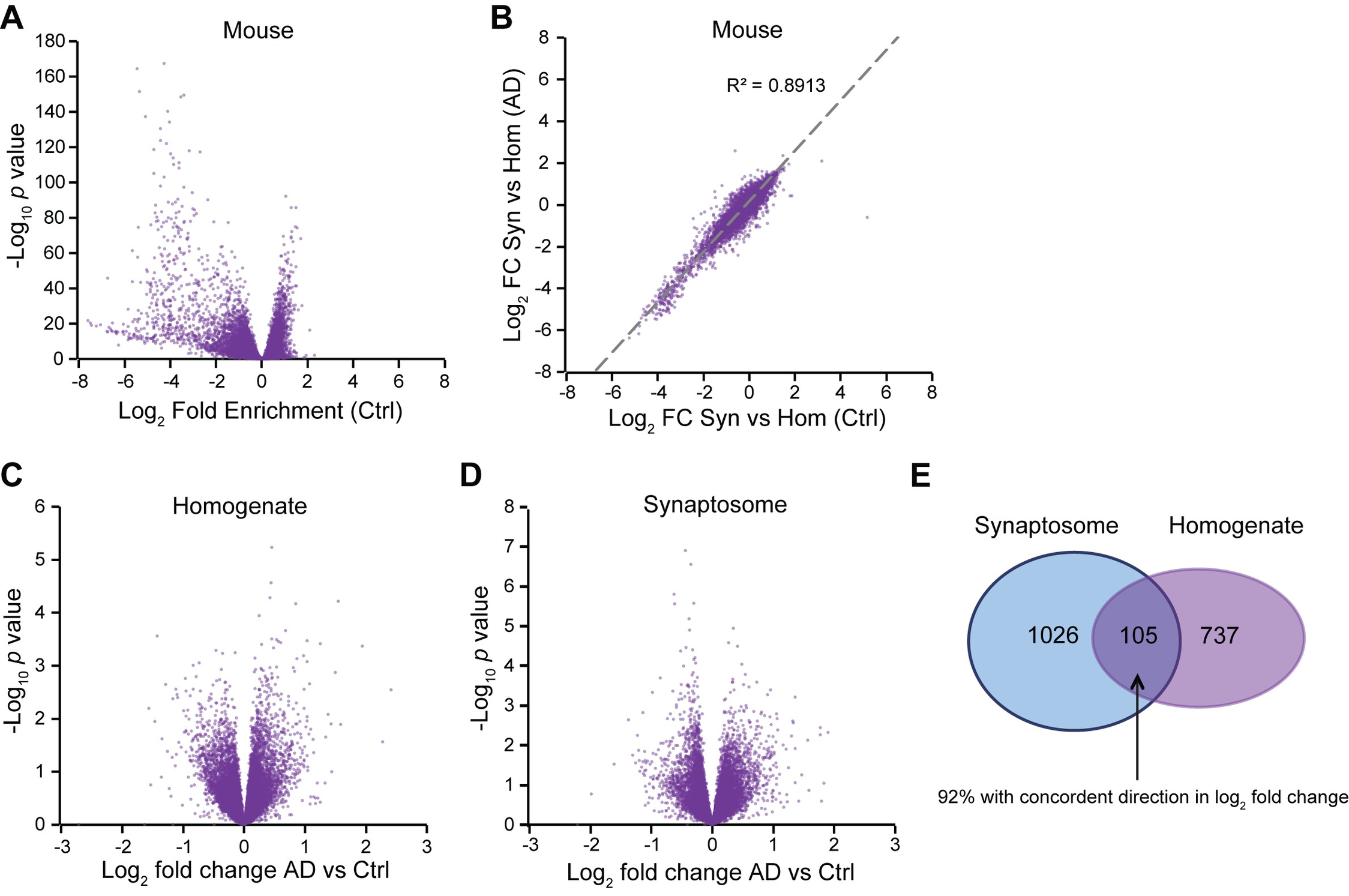

Supplement: S9 Fig — A. Volcano plot showing synaptosome-enriched mRNAs in control mouse frontal lobe samples. B. Comparison of synaptosome-enrichment (p < 0.01) between AD and control mouse frontal lobe samples shows strong correlation (R2 = 0.8913). C. Volcano plot comparing expression differences between AD and control in the bulk, unfractionated homogenate. D. Volcano plot comparing expression differences between AD and control just within the synaptosome particles. E. Venn diagram showing minimal overlap of differentially expressed mRNAs in mouse frontal lobe synaptosome and homogenate fractions (p < 0.05). Those that do overlap have 92% concordant log2 fold change. (TIF) [file pgen.1011359.s009.tif]

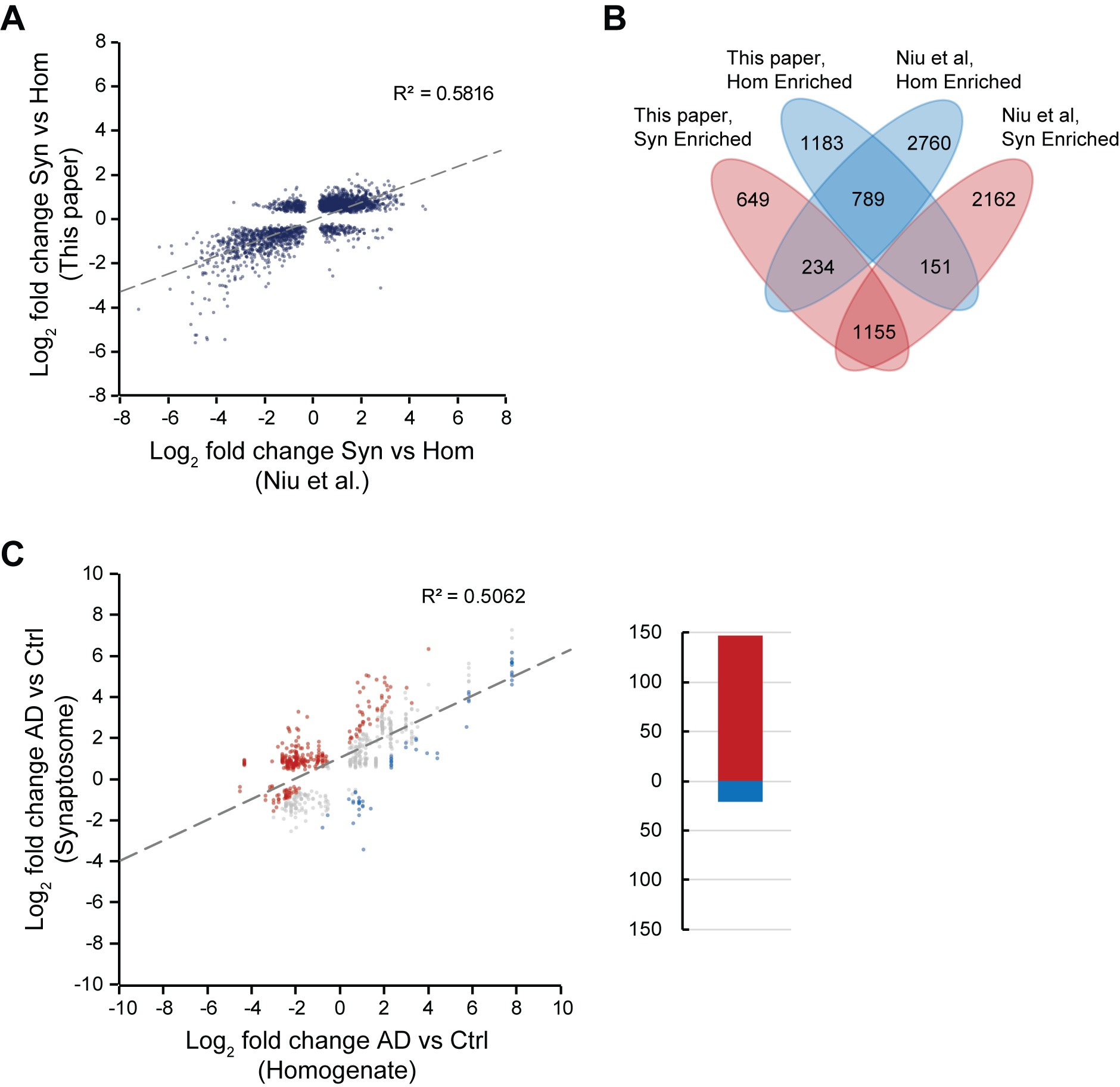

Supplement: S10 Fig — A. Comparing the fold changes/enrichment of synaptosome vs homogenate between our data and Niu et al. shows a moderate correlation (R2 = 0.5816). B. Venn diagram shows plenty of synaptosome enriched genes overlap between our data and Niu et al. C. Comparing the fold changes of AD vs control between synaptosomes and homogenate in Niu et al.’s data shows moderate correlation (R2 = 0.5062). Genes that have a T-statistic most distant from a y = x trendline are significantly mislocalized, independent of global expression changes (p < 0.05). (TIF) [file pgen.1011359.s010.tif]

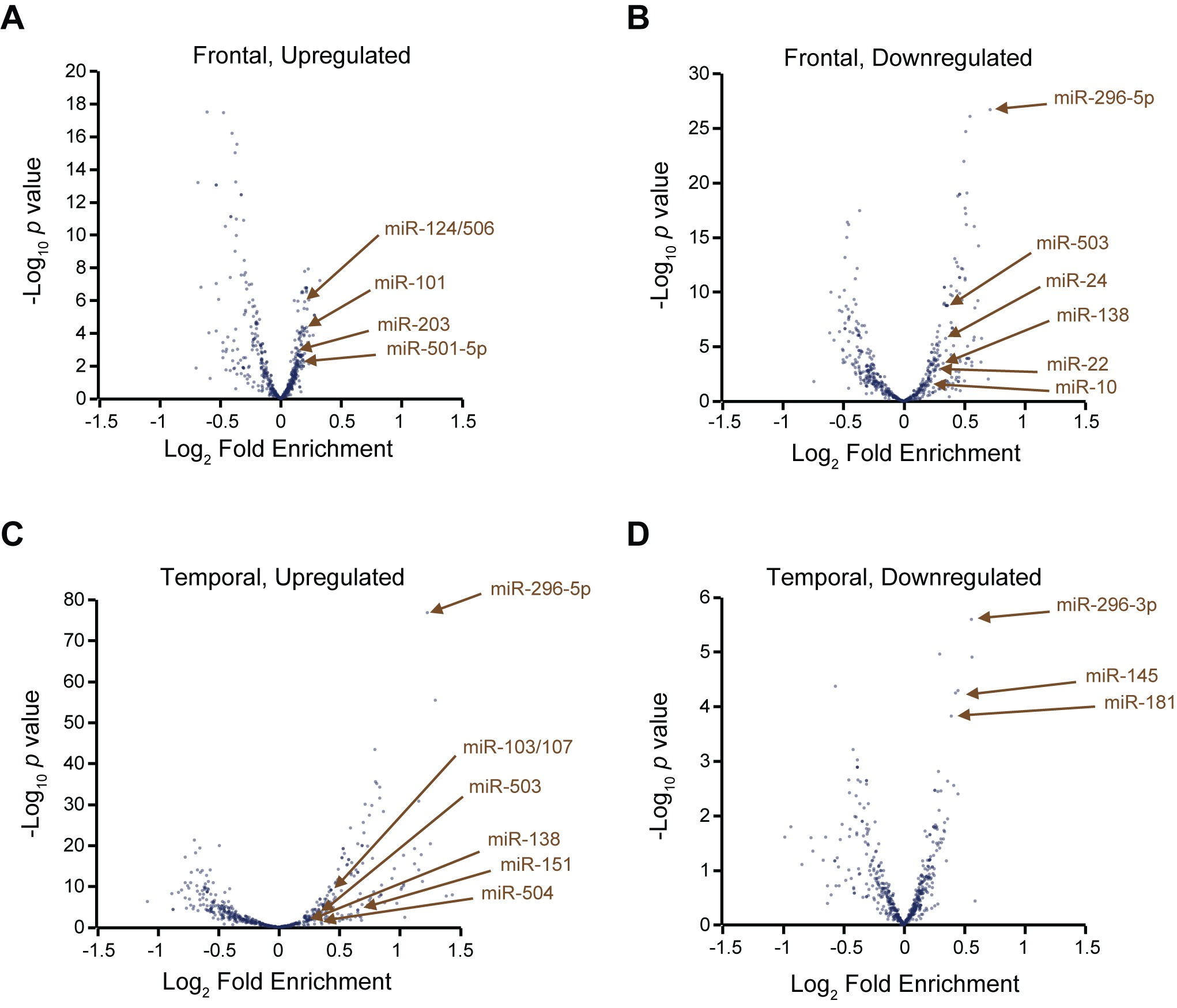

Supplement: S11 Fig — A. Volcano plot showing enrichment of microRNA binding sites among the upregulated mislocalized mRNAs in the human frontal lobe (p < 0.05, see Fig 2D). B. Volcano plot showing enrichment of microRNA binding sites among the downregulated mislocalized mRNAs in the human frontal lobe (p < 0.05, see Fig 2D). C. Volcano plot showing enrichment of microRNA binding sites among the upregulated mislocalized mRNAs in the human temporal lobe (p < 0.05, see Fig 3D). D. Volcano plot showing enrichment of microRNA binding sites among the downregulated mislocalized mRNAs in the human temporal lobe (p < 0.05, see Fig 3D). (TIF) [file pgen.1011359.s011.tif]

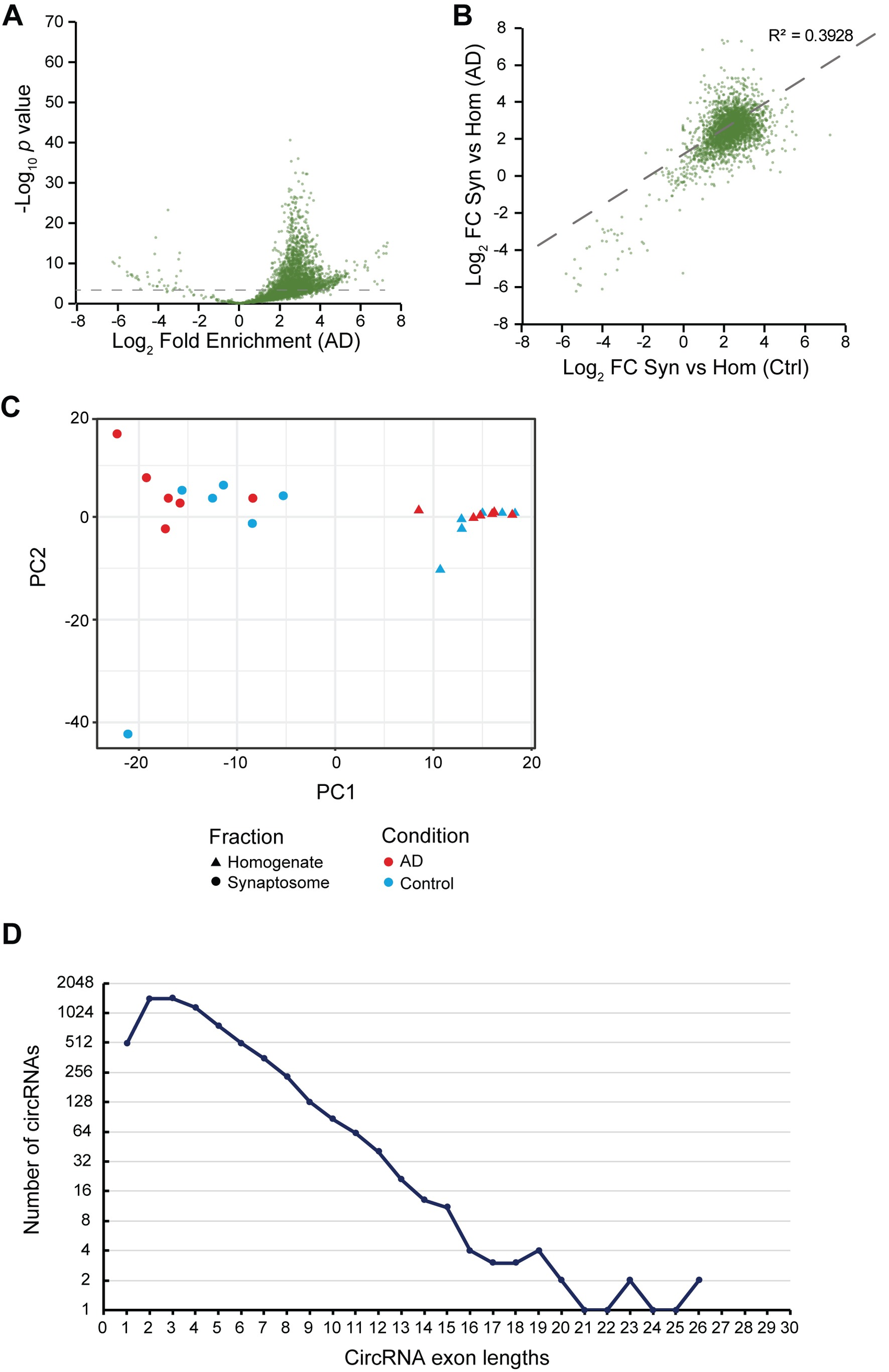

Supplement: S12 Fig — A. Volcano plot comparing circRNAs in synaptosome vs homogenate in AD frontal lobe samples shows that the majority of circRNAs are significantly enriched at synaptic terminals. B. Comparing circRNAs in synaptosome vs homogenate in both AD and control frontal lobe samples shows that synaptic enrichment of circRNAs is a shared phenomenon. C. Multidimensional scaling plot of samples used for circRNA analysis representing the variation exclusively among circRNA reads (this is separate from the differential expression analysis where the combination of linear and circular reads were used for normalization). D. Analyzing the difference between the donor and acceptor exons involved in back-splicing shows that the majority of circRNAs consist of 2–3 exons. (TIF) [file pgen.1011359.s012.tif]

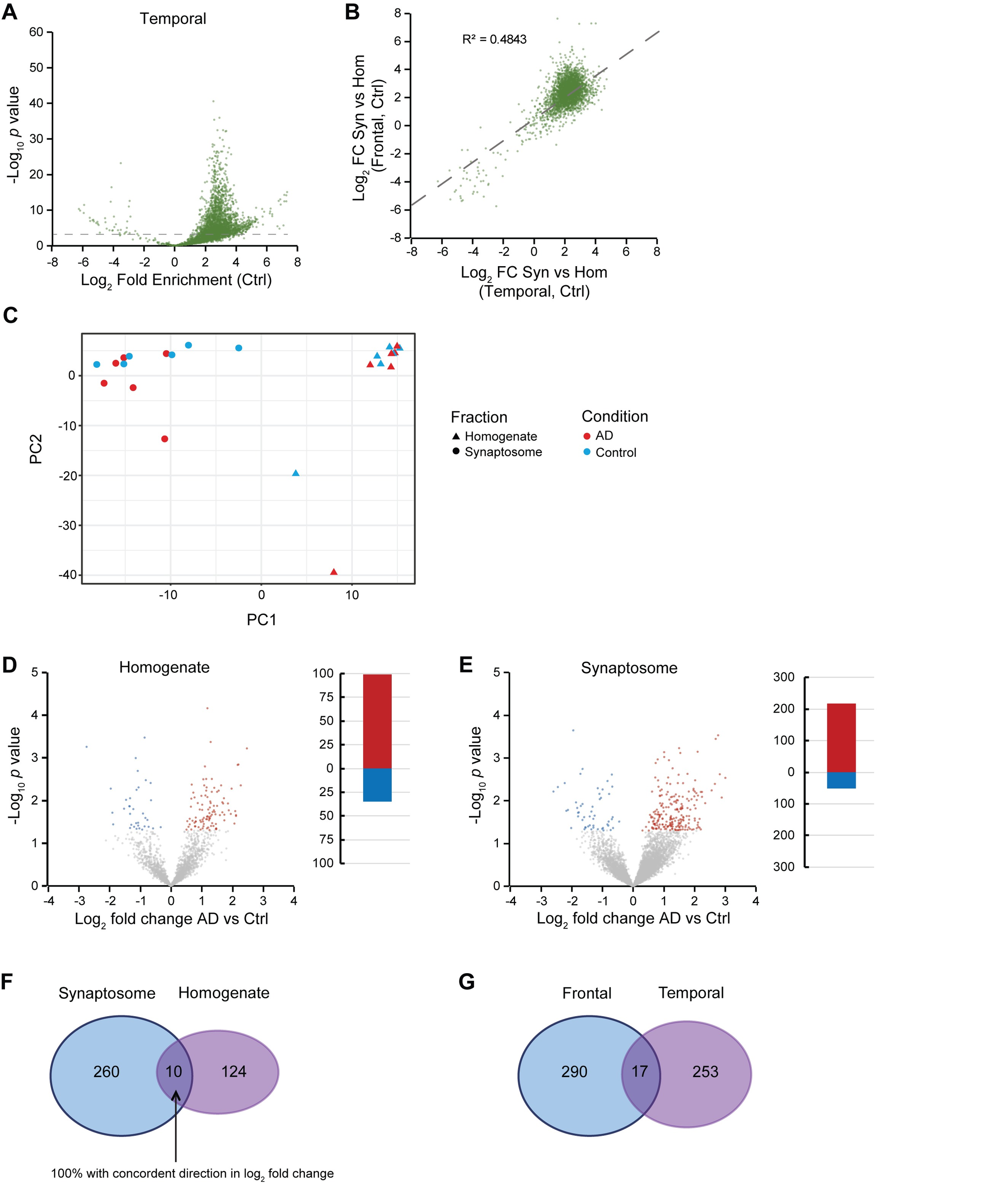

Supplement: S13 Fig — A. Volcano plot comparing circRNAs in synaptosome vs homogenate in control temporal lobe samples shows that the majority of circRNAs are significantly enriched at synaptic terminals. B. Comparing circRNAs in synaptosome vs homogenate in both control frontal lobe and temporal lobe samples shows that synaptic enrichment of circRNAs is similar in both tissues. C. Multidimensional scaling plot of samples used for circRNA analysis representing the variation exclusively among circRNA reads (this is separate from the differential expression analysis where the combination of linear and circular reads were used for normalization). D. Volcano plot comparing circRNAs in AD vs control in the unfractionated homogenate (p < 0.05). E. Volcano plot comparing circRNAs in AD vs control in the synaptosome fraction (p < 0.05). F. Venn diagram shows minimal overlap of differentially expressed circRNAs between synaptosome and homogenate fractions with more occurring in the synaptosome fraction (p < 0.05). Those that do overlap show 100% concordant Log2 fold change. G. Venn diagram shows minimal overlap of differentially expressed circRNAs in the synaptosome fraction between human frontal and temporal lobes (p < 0.05). (TIF) [file pgen.1011359.s013.tif]

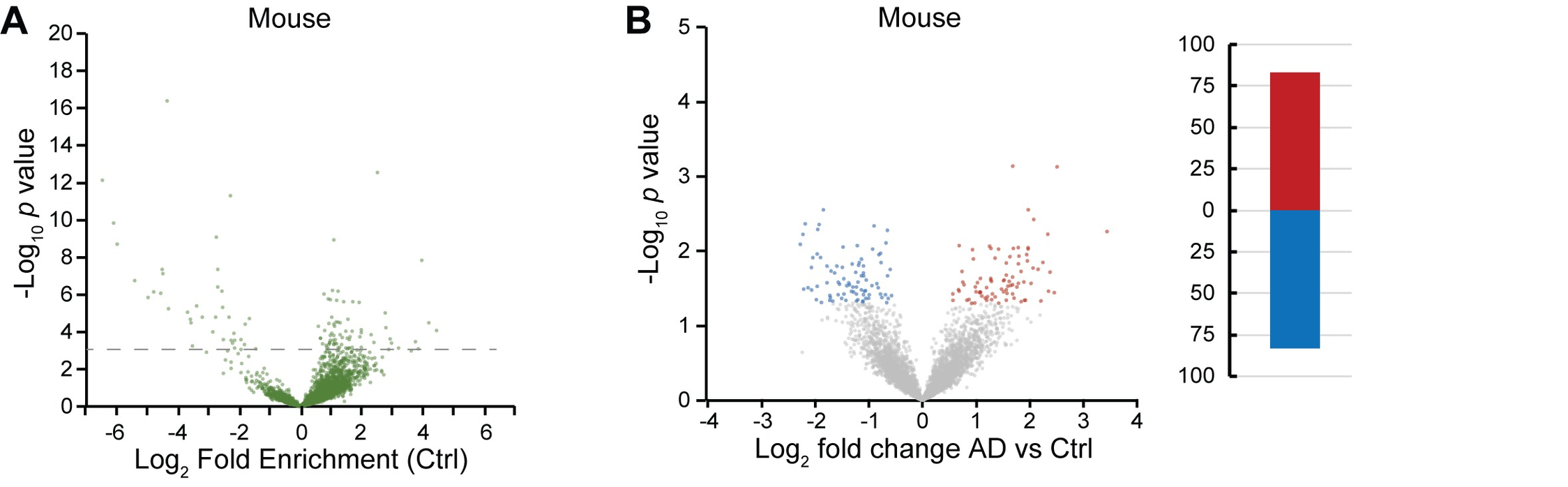

Supplement: S14 Fig — A. Volcano plot comparing circRNAs in synaptosome vs homogenate in control mouse samples continues to show synaptic enrichment of circRNAs. B. Volcano plot comparing circRNAs in AD vs control in mouse synaptosomes (p <0.05). (TIF) [file pgen.1011359.s014.tif]

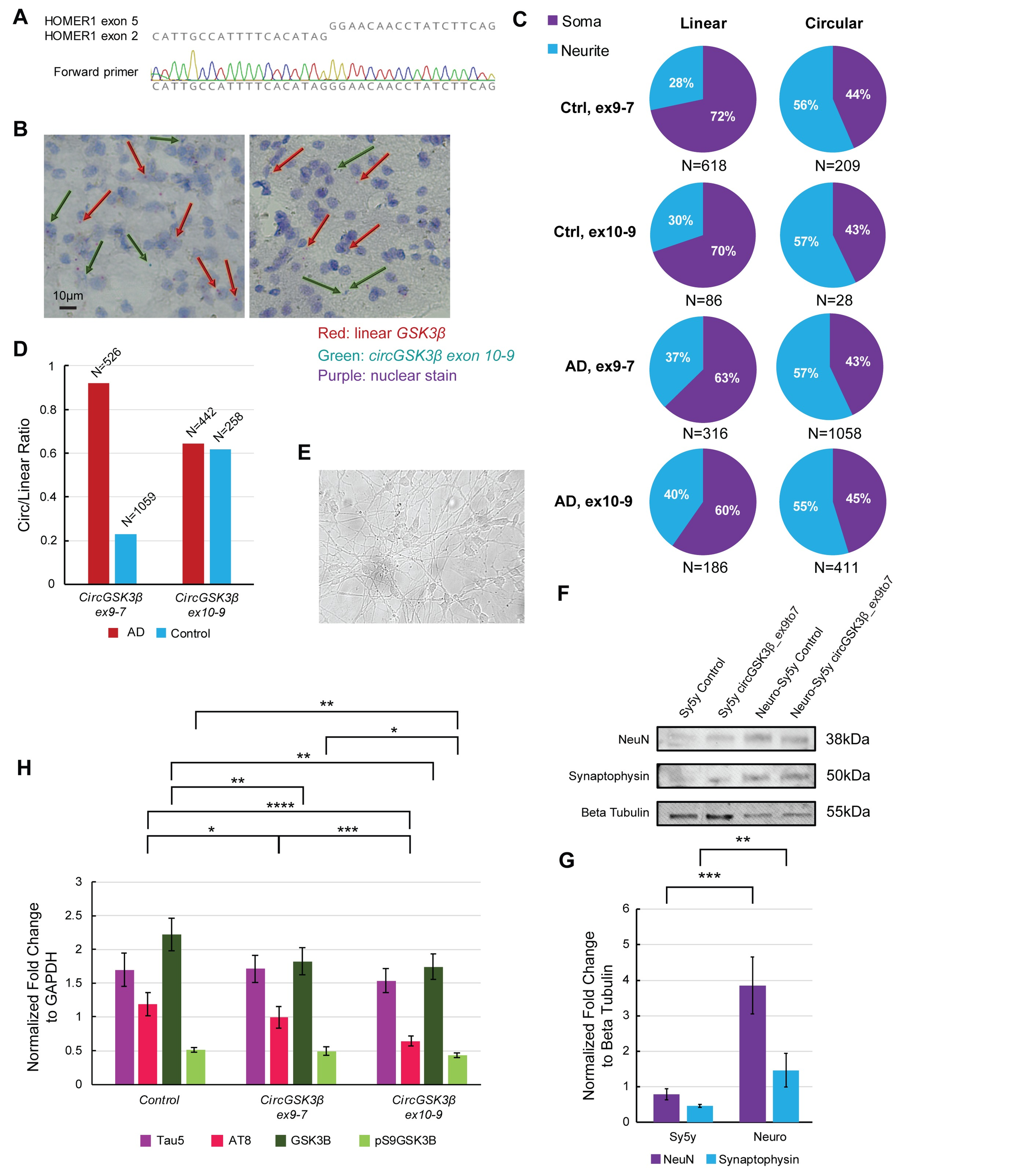

Supplement: S15 Fig — A. Sanger Sequencing confirmation of back-splice junction between exons 5 and 2 of HOMER1. B. BaseScope RNA hybridization probes show that circGSK3β isoforms (green) are more frequently distal to the nucleus than the linear GSK3β transcript (red) in human control frontal lobe brain slices. Tissue and nuclei were stained with hematoxylin. C. Pie charts representing quantification of nuclear versus non-nuclear localization of linear GSK3β and circular isoforms in BaseScope images (not significant). D. Quantification of GSK3β circular isoforms in AD vs control in BaseScope images (not significant). E. Bright field microscopy of neuron-differentiated SH-Sy5y cells confirms development of neuron morphology including polarized cells, axons, and dendrites. F. SH-Sy5y neuron differentiation confirmed by western blot showing an enrichment of two neuronal markers, Synaptophysin and NeuN. G. Quantification of blots in (S15F Fig). N = 6 samples per group. **: p < 0.01, ***: p < 0.001. H. Quantification of blots in (Fig 6E). N = 8 samples per group Significance calculated by T-test. *: p < 0.05, **: p < 0.01, ***: p < 0.001, ****: p < 0.0001. (TIF) [file pgen.1011359.s015.tif]

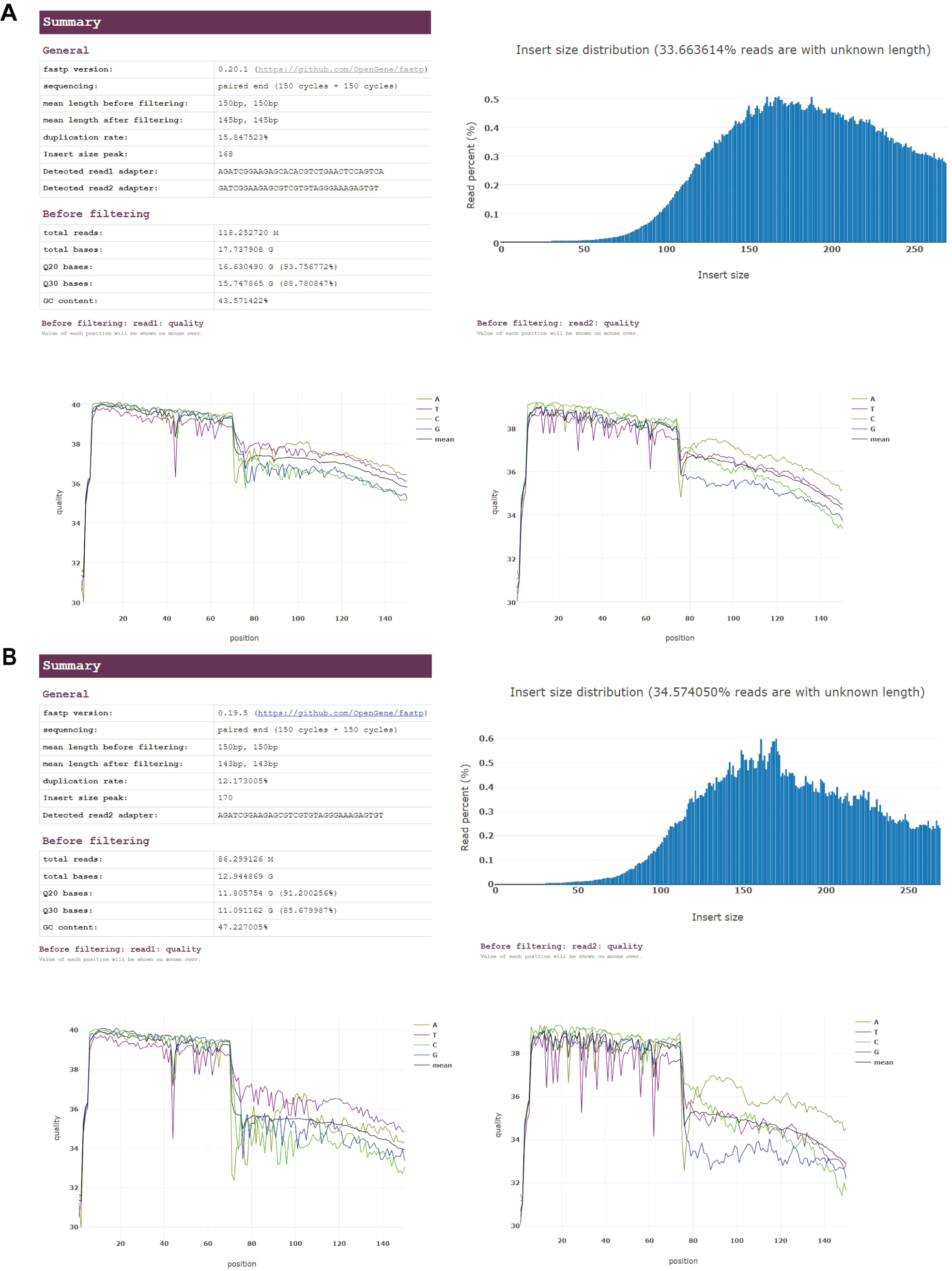

Supplement: S16 Fig — Screenshots from the output of quality assessment tool, Fastp, found on the Galaxy online resource. A. Representative quality assessment of a homogenate sample. B. Representative quality assessment of a synaptosome sample [95]. (TIF) [file pgen.1011359.s016.tif]

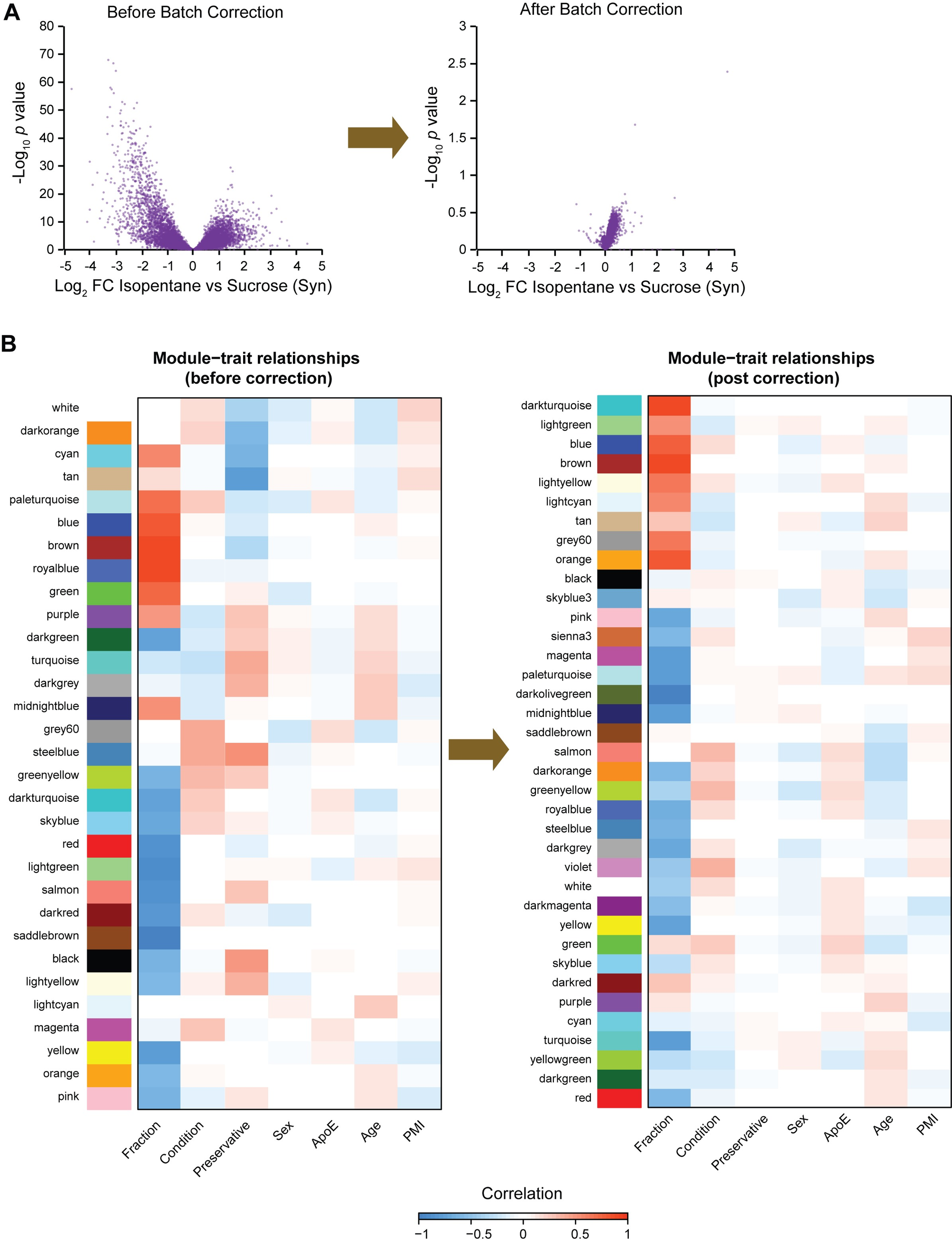

Supplement: S17 Fig — A. Volcano plots comparing isopentane vs sucrose tissue preservation among both AD and control frontal lobe synaptosome samples before and after batch correction. ComBat_seq appears to substantially reduce the variation among samples based on this variable [87]. B. Weighted gene correlation network analysis (WGCNA) among human AD and control frontal lobe homogenate and synaptosome samples identifies confounding variables before and after ComBat_seq batch correction which shows that batch correction almost completely removes gene expression modules that correlate with preservation method with little impact on the expression variation defined by modules correlated with other variables including fraction and condition. (TIF) [file pgen.1011359.s017.tif]
